# The effects of Mid-Holocene foragers on the European oyster in Denmark

**DOI:** 10.1073/pnas.2410335121

**Published:** 2024-10-28

**Authors:** Harry K. Robson, Niklas Hausmann, Eva M. Laurie, Peter Moe Astrup, Karen Povlsen, Søren A. Sørensen, Søren H. Andersen, Nicky Milner

**Affiliations:** ^a^Department of Archaeology, University of York, York YO10 5DD, United Kingdom; ^b^Leibniz Zentrum für Archäologie, Mainz 55116, Germany; ^c^Moesgaard Museum, Højbjerg 8270, Denmark; ^d^The Historical Museum of Northern Jutland, Algade 48, Aalborg, Denmark; ^e^Roskilde Museum, Roskilde 4000, Denmark

**Keywords:** prehistoric baseline, archaeological shellfish, hunter-gatherer-fishers, southern Scandinavia, paleoecology

## Abstract

Oysters (Ostreidae) play a pivotal role in the health and productivity of marine ecosystems. Their unique ability to filter water, provide habitat, and contribute to nutrient cycling has remained underused in many parts of Europe following the destruction of vast oyster beds in the 19th and 20th centuries. The burgeoning field of oyster restoration for aquaculture has recognized the potential of these bivalves in promoting ecosystem resilience and enhancing biodiversity. Restoring oysters to previous levels requires the establishment of ecological baselines that ideally take into account the long-term changes of animal behavior as well as the surrounding environment prior to significant human intervention, an extremely challenging task. Archaeological shell middens are invaluable baseline archives and provide exclusive insights into past ecosystems. Here, we use demographic information from over 2,000 analyzed European oyster (*Ostrea edulis*) shells dating from ~5,660 to 2,600 cal BCE (calibrated years BCE), the largest archaeological growth rate dataset of mollusks yet. Through the analysis of size as well as ontogenetic age, we decouple anthropogenic from environmental impacts throughout Denmark. Our data show definitive influence of oyster size–age structure through human harvesting during the Mid-Holocene, with older oysters in the Mesolithic (mean: 4.9 y) than the Neolithic (mean: 3.7 y), irrespective of changes in growth rate. Furthermore, we present the metrics for long-term sustainable harvesting of oysters across environmental and socioeconomic transitions, providing demographic targets for current oyster restoration projects and valuable context in mitigating the impact of modern climatic change.

## Significance

This study examines the long-term influence of human harvesting throughout Denmark. By analyzing growth rate changes, we better quantify the pivotal role of harvesting pressure in prehistoric marine ecosystems. Modern European baselines refer to depleted oyster populations as a result of overharvesting during the 19th and 20th centuries. Dating from ~7,660 y ago, this study’s data predate industrial large-scale oyster fishing and demonstrate that long-term sustainable oyster fishing was practiced at almost all of the sampled archaeological sites, providing a unique and important reference for modern conservation efforts with implications for future aquaculture practices and governance. Our findings further underscore the importance of preserving these keystone species to maintain the health and balance of marine habitats.

## Biological and Economic Functions of Oysters

Oysters (Ostreidae), as ecosystem engineers, are able to significantly influence the ecological dynamics and economic sustainability in coastal regions worldwide (1, 2), particularly estuarine areas, enhancing the seafood industry and ecotourism, offering additional revenue for local communities (3, 4). The pivotal role of oysters in these ecosystems is primarily due to their robust filtration capacity, serving as an organic particle and pollutant extractor. This process improves water transparency, encourages aquatic plant photosynthesis, and curbs eutrophication by limiting nitrogen and phosphorus, thereby maintaining a biologically diverse and healthy marine environment (5). Moreover, the tendency of oysters to establish reefs contributes to our understanding of ecological complexity and biodiversity by providing crucial habitats for various marine species. The physical presence of reefs also mitigates shoreline erosion and acts as a storm buffer, which is increasingly important considering modern climate change (6). The vital ecological and economic roles of oysters necessitate strategic conservation and sustainable management efforts to preserve their ecological functions and ensure the longevity of supported human industries, thus balancing ecological health with human well-being.

## Overfishing, Restoration Efforts, and Challenges in Establishing Accurate Baselines

Historically abundant oyster populations have considerably declined globally due to overfishing, pollution, habitat degradation, diseases, Allee effects, pests and parasites, and climate change (7). The decline of oyster populations in the Pacific and particularly along the European and American Atlantic coasts has resulted in significant ecological and economic concern (8–10). Similar issues are evident in the North Sea where industrial-scale harvesting throughout the 19th and early 20th centuries has critically reduced North Sea oyster (*Ostrea edulis*) populations by 90% [*SI Appendix*, Fig. S1; (11, 12)]. A century ago, complex, three-dimensional habitats in the Wadden Sea (southeast North Sea) were provided by oyster banks (11). These diversified the region’s underwater landscape and provided a range of habitats for a variety of species which depended on hard substrates for protection or food supply, including rich communities of sponges, sea anemones, hydroids, worms, sea urchins, and others (13, 14). With the oysters’ depletion, the Wadden Sea ecosystem’s ability to provide high water quality and complex habitats was hugely reduced, leading to the tidal flats we know today (15, 16). Climate change has intensified these effects through elevated sea temperatures, ocean acidification, and frequent extreme weather events (17).

In light of these ecological challenges, several restoration and repopulation efforts are currently underway. In Europe, the Native Oyster Restoration Alliance (NORA) (1) and the European Union’s Horizon 2020 program have initiated projects aimed at rebuilding lost oyster reefs, and enhancing the populations of native oysters (*O. edulis*) in these regions (e.g., ref. 18). Elsewhere, for instance, along the American Atlantic coast, a number of similar initiatives are being undertaken, such as the Billion Oyster Project (19) and the Chesapeake Bay Native Oyster Recovery Program (20–22). However, both historical and ongoing stresses have altered the modern baseline of oyster populations. Baselines refer to an undisturbed state of an ecosystem, against which current conditions can be compared to assess human-induced changes. This shift means that our perception of a healthy oyster population is potentially based on a significantly depleted standard (8, 23). Restoration targets that enhance ecosystem functions and services, such as biodiversity, habitat complexity, and water quality, are contingent upon a comprehensive understanding of oyster fisheries before historical overfishing occurred.

## Research on Prehistoric and Historic Oyster Fisheries

Progressive environmental degradation can be a problem for marine ecosystems because the history of marine ecosystem change is largely unknown, points of reference forgotten or overprinted, leading to a gradual acceptance of the degradation (23). To address this knowledge gap, researchers are increasingly turning to paleoecological, archaeological, and historical evidence (24). These studies are revealing the timing, direction, magnitude, and drivers of change within marine ecosystems over past decades, centuries, and millennia. For example, comparing historical (1878–1935) and recent (1968–2010) records, zu Ermgassen et al. (25) revealed a 64% decline in the extent of eastern oyster (*Crassostrea virginica*) habitats, and an 88% decrease in biomass in US estuaries. Lotze (11) summarizes the historical data (1770–1930) on European oyster (*O. edulis*) fishing in the Wadden Sea near Jutland, Denmark, showing how annual oyster landings dramatically fluctuated from less than 100,000 to more than 5 million in the 1860s, before dropping to below 1 million by the 1880s until their eventual collapse during the mid-20th century. Currently, the largest natural population of *O. edulis* in the North Sea basin is found in the Limfjord of Denmark, while smaller and endangered natural populations are known in Belgium, the Netherlands, Norway, and Sweden (26, 27). Using survey data and extensive dating of shell middens along the Dornoch Firth and Moray Firth systems, Scotland, Fariñas-Franco et al. (28) provide evidence for the prehistoric and historic presence of the European oyster (*O. edulis*). Their findings suggest a persistent *O. edulis* population in these areas until the 19th century. Archaeological oysters also provide metric information on past ecologies (29). For instance, oyster sizes can be used as a proxy for examining the conditions of past coastal ecosystems and for understanding the stability and resilience of oyster fisheries over time (30). Rick et al. (31) used eastern oyster (*C. virginica*) shell sizes from Chesapeake Bay to show that Native American fisheries were focused on nearshore oysters and were likely harvested at a rate that was sustainable for ~3,500 y, despite changing Holocene climatic conditions and sea-level rise. Similarly, Thompson et al. (32) concluded that *C. virginica* fisheries remained resilient with multiple stable states over the last 5,000 y before the 1900s, despite demographic and socioeconomic shifts.

The case studies above illustrate how archaeological information can provide an important perspective and substantial amounts of first-hand measurable information on the long-term development of oyster fishing across millennia and through changing environmental conditions.

While oyster sizes are usually implied to be mainly affected by human harvesting pressure, the caveat of environmental changes as an additional factor is also included (31–33). However, few attempts are made to quantify how much each factor (harvesting pressure and environmental changes) actually affects size (30, 34). By adding the biological age of oysters as a measure, one can observe whether changes in their size are due to natural environmental changes or human activities (35). Similar to tree rings, oyster shells have regular markers that help us determine their age (36). If we find that both small and large shells are about the same age, it might mean that changes in the environment (e.g., changes in salinity, temperature, or nutrient availability) are causing the oysters to grow more slowly and stay small. However, if we find that the oysters as a whole are getting smaller over time and are also being harvested at a younger age, it could mean that people are overharvesting. When people harvest oysters heavily, they often take the larger, older ones or remove the younger ones before they can grow large (37). This results in the oyster population as a whole being both younger and smaller (38), with subsequent negative effects on the overall fertility of the population as older oysters disproportionately outperform younger ones (39).

Since environmental changes have been shown to have a strong control on shellfish populations, it is crucial to quantify their influence, when using their paleoecological information to determine a long-term baseline. For instance, Toniello et al. (35) used the ages and growth rates of 124 butter clams (*Saxidomus gigantea*) to investigate the historical ecology of these species in the Salish Sea, British Columbia, integrating archaeological, paleoecological, and contemporary ecological records spanning 11,500 y. The study revealed that the sizes and lifespans of these clams increased over time owing to favorable environmental conditions and human cultivation practices, but posited that the clams in the present day are less productive due to altered habitats and the absence of traditional practices.

Being able to decouple both factors in the interpretation of oyster size further improves our understanding of the sustainable use of oyster beds by preindustrial coastal populations and emphasizes their role as ecological archives in the guidance of modern economic uses of oysters.

## Long-term Demographic Data

**Fig. 1.**
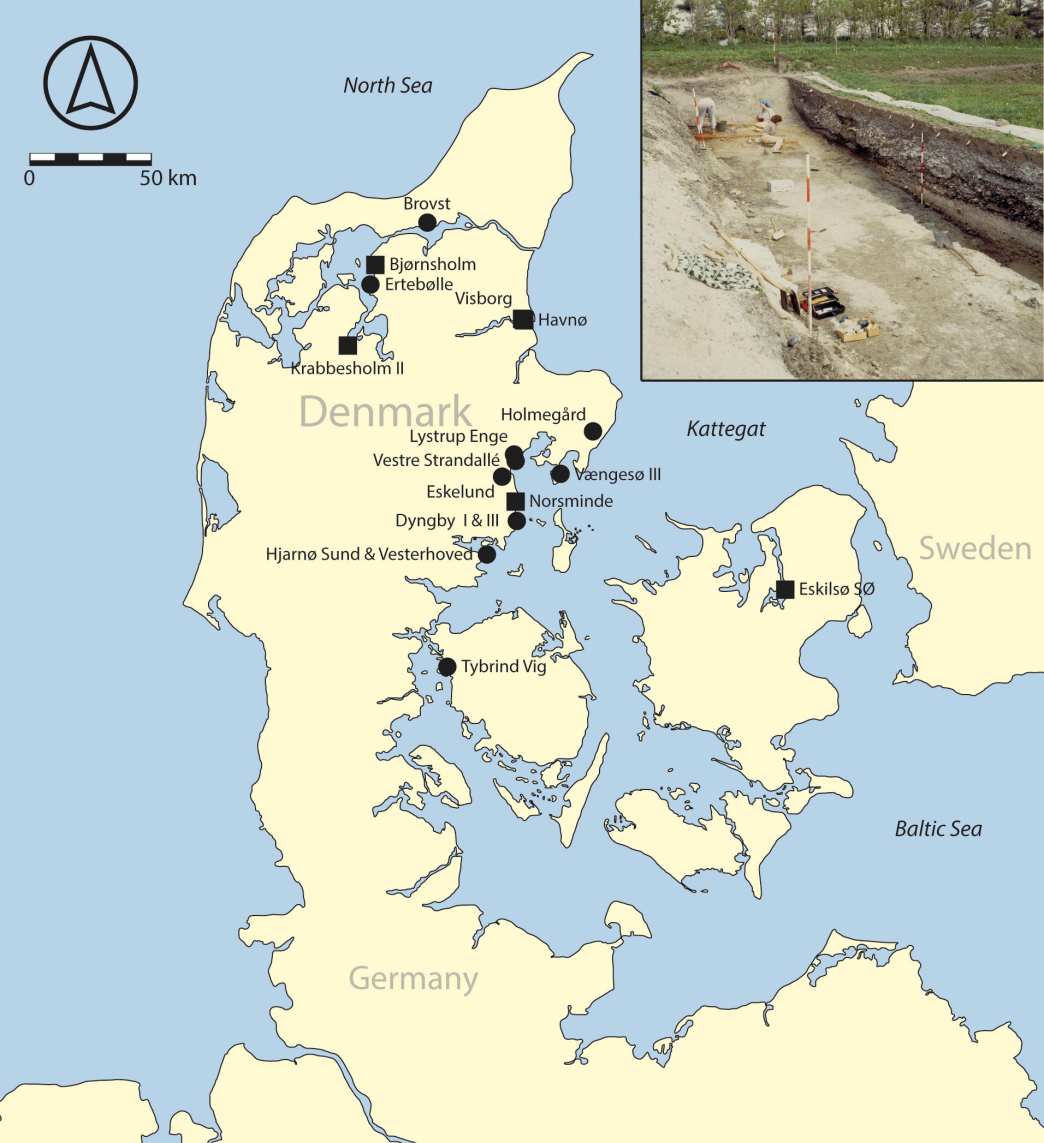
The natural shell bank (i.e., shell deposit at Tybrind Vig) and archaeological sites sampled in this study, including those with Middle–Late Mesolithic (circle) or Late Mesolithic and Early Neolithic (square) oysters. The inset shows one of the trenches through the eponymous Ertebølle shell midden (base-map by Vemaps, https://vemaps.com/denmark/dk-05).

Here, we present a comprehensive summary of the largest archaeological demographic dataset of mollusks to date, featuring data from over 2000 European oyster (*O. edulis*) specimens and spanning a ~3000-year period (~5,660 to 2,600 cal BCE). The dataset covers 19 archaeological sites and a natural shell bank across Denmark (Fig. 1 and *SI Appendix*, Table S1), including areas where wild *O. edulis* are still available, as well as areas where prehistoric coastlines have been submerged. Several of the sites are located in the Limfjord (i.e., Bjørnsholm, Brovst, Ertebølle, Krabbesholm II), which was connected to the North Sea throughout prehistory (40). With the exception of Eskilsø, located in Roskilde Fjord, Zealand, and Tybrind Vig, situated on the coastline of north-west Fyn, the remaining archaeological sites are located on the eastern coastline of Jutland (Fig. 1). Throughout prehistory when Jutland was an archipelago, saline waters from the North Sea inflowed, creating ideal conditions for the formation of shell beds throughout Denmark (40). For each shell we aimed to determine the size as well as its age at death through sclerochronology, providing us with the additional measure of its growth rate (*Materials and Methods*).

In addition to its spatially extensive character, the dataset covers environmental fluctuations occurring during the Mid-Holocene and socioeconomic shifts in the form of the Mesolithic-Neolithic transition. Our dataset primarily covers the Late Mesolithic Ertebølle culture, which was distributed across present-day Denmark, parts of northern Germany and southern Sweden (41). This period is characterized by semisedentary hunter-gatherer-fishers, with communities living along dynamic coastlines and specializing in the exploitation of marine resources. A stand-out feature of the Ertebølle culture in Denmark is the presence of numerous shell middens throughout the north and north-west of the country (Fig. 1) (42); large accumulations of shell debris interspersed with settlement refuse, including, for instance, antler, bone and stone tools, botanical and faunal remains, ceramics, as well as structures such as dwellings, hearths, and pits (42). While these unique archaeological sites have contributed substantially to our understanding of the culture, there are thousands of nonmidden coastal and inland sites, which have similarly been informative. Transitioning into the Neolithic period at 4,000/3,900 cal BCE, the Funnel Beaker culture became widespread across much of Northern Europe, including present-day Denmark, Germany, and parts of Sweden, and as far east as the Vistula River in Poland. Named after the characteristic shape of its pottery, this culture marked the advent of farming in Denmark. Although marine resources, including shellfish, continued to be exploited, there was a noticeable shift toward animal husbandry and crop cultivation (43).

Our dataset also covers dynamic environmental conditions with changes in sea-level, water-inflow from the North to the Baltic Sea, and marine transgressions which likely impacted the Danish *O. edulis* population. During the Mid-Holocene, European oysters were, in some cases, replaced by common cockles (*Cerastoderma edule*) and blue mussels (*Mytilus edulis*) among other taxa (44, 45)—a phenomenon often referred to as the “oyster-to-cockle” shift. At many sites, this shift is thought to coincide with the Mesolithic-Neolithic transition and a number of different possible explanations have been suggested, including environmental and cultural factors (37, 38). Despite this general pattern, there is considerable evidence for the continued exploitation of *O. edulis* well into the Neolithic (e.g., ref. 42), demonstrating that the phenomenon was not homogenous throughout the region.

Our study aims to investigate the dynamics of the prehistoric oyster population in Denmark and to quantify the influence of age and environmental factors across a large number of sites. Despite some suggestions otherwise (46), we hypothesize that the Neolithic period, characterized by an increased reliance on an agricultural subsistence economy and reduced harvesting pressure, would lead to an increase in the age of *O. edulis* at most sites. Furthermore, the oyster-cockle-shift could be grounded in environmental changes or nonsustainable harvesting pressure that affected oysters in a way that favored *C. edule* as dominant species. We would then expect a decrease in oyster sizes prior to this shift.

This research underscores the significance of historical oyster population dynamics, offering insights that are crucial for developing future conservation strategies to sustain healthy oyster beds and lay a foundation for a better understanding of the interaction between environmental factors and sustainable harvesting practices.

## Results

In total, we analyzed 2,107 European oyster (*O. edulis*) shells from 19 archaeological sites and a natural shell bank throughout Denmark. Of these, we obtained data on hinge size and/or biological age in 1990 shells [Table 1 and *SI Appendix*, Table S1 and this article’s Open Science Framework data repository (47)]. For 143 shells it was not possible to measure the hinge size and for 683 it was not possible to determine the biological age. The sample size for each of the archaeological sites is shown in *SI Appendix*, Fig. S2*A* together with their distributions across archaeological periods and measurement type (*SI Appendix*, Fig. S2*B*). The number of samples is not correlated to the size of each sampled site but is a result of preservation, research intensity, excavation methods, and accessibility to the authors (*Materials and Methods*).

**Table 1.** Summary metrics of shells

	Minimum	1st Quartile	Median	Mean	3rd Quartile	Maximum
Hinge in mm	1.3	6.0	7.6	8.2	9.5	33.4
Length^*^ in mm	9.3	63.4	71.7	74.3	79.6	124.1
Age in years	1.0	2.0	3.0	4.5	5.0	23.0

^*^Length of oyster shells is determined via an empirical equation based on Milner (48) (*Materials and Methods* and *SI Appendix*, Fig. S5).

### Sizes.

**Fig. 2.**
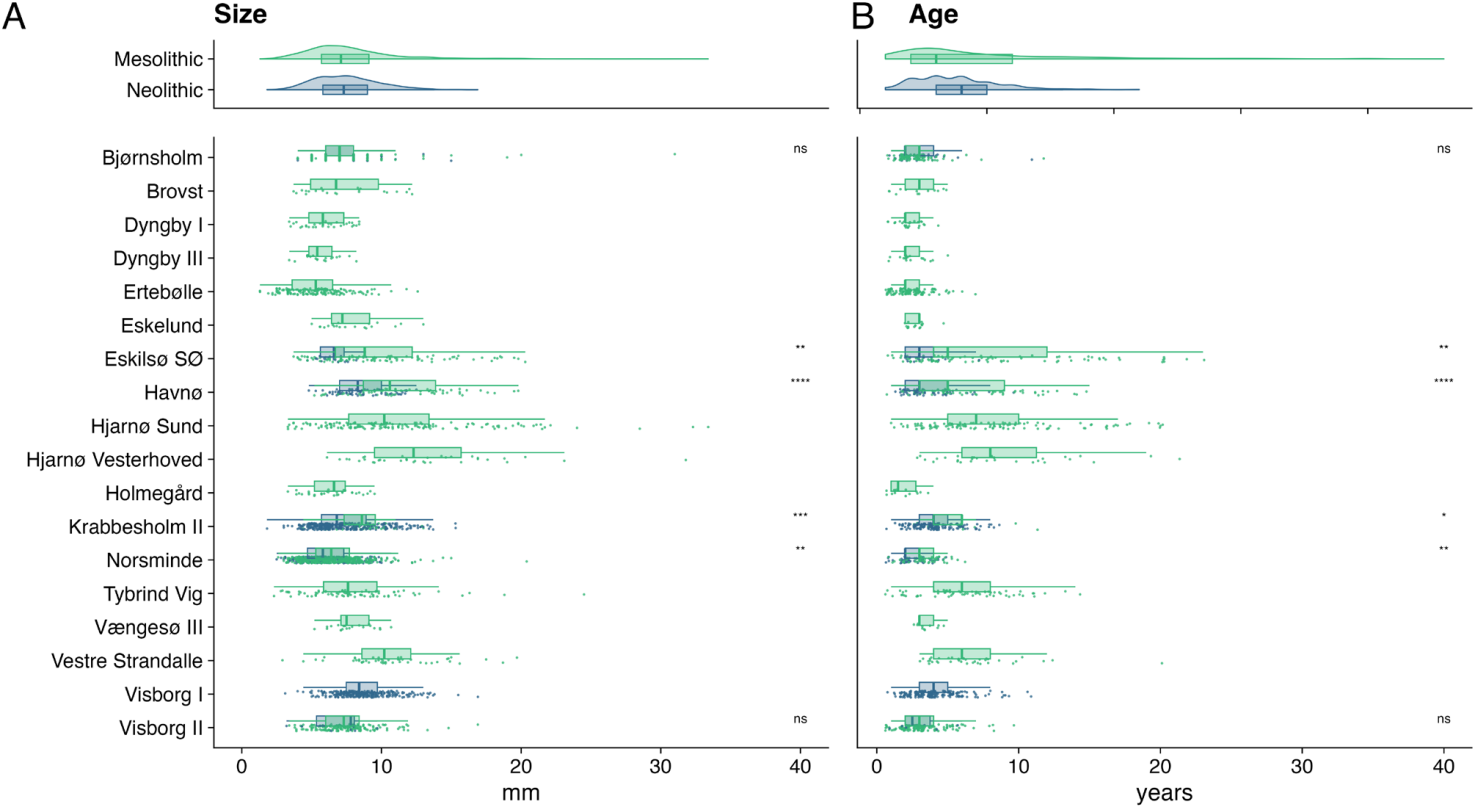
(*A*) Size distribution by period and archaeological site. Length of oysters calculated based on hinge measurements. (*B*) Age distribution by period and archaeological site. Stars (*) indicate significant differences between Mesolithic and Neolithic distributions following Mann–Whitney *U* tests with increasing significance for each additional star (*P < 0.05, **P < 0.01, and ***P < 0.001); “ns” indicates no significant difference.

There were changes in the size of oysters between periods with Mesolithic shells having a significantly wider range of hinge sizes (2 to 34 mm, range = 32 mm) than the Neolithic oysters (2 to 18 mm, range = 16 mm) (F test, *P* < 0.001; Levene’s test, *P* < 0.001) (Fig. 2*A*). This translates into shell lengths of ~1.5 to 14.0 cm (mean: 7.5 cm, median: 7.1 cm, range = 12.5 cm) for the Mesolithic and ~1.5 to 10.9 cm (mean: 7.4 cm, median: 7.3 cm, range = 9.4 cm) for the Neolithic shells. The average hinge size of the Mesolithic shells is larger (8.3 mm, n = 1225) than the Neolithic ones (7.9 mm, n = 739) (Fig. 2*A*), but not significantly (Mann–Whitney *U* test, *P* = 0.22) and only as a result of some Middle-Late Mesolithic sites such as Hjarnø Sund and Hjarnø Vesterhoved skewing the overall values (see Fig. 2*A*). Comparisons between the periods on a site level were possible at six of the 20 sites (Fig. 2*A*). The pooled size measurements for all Mesolithic shells were significantly larger than those from the Neolithic deposits at four of the six sites (Mann–Whitney *U* test; Krabbesholm II: *P* < 0.01; Havnø: *P* < 0.01; Eskilsø SØ: *P* < 0.01; Norsminde: *P* < 0.01). At two sites there was no significant difference (Mann–Whitney *U* test; Bjørnsholm: *P* = 0.70; Visborg: *P* = 0.91) to be found (Fig. 2*A*).

### Ages.

We further compared biological ages between periods. Our results show that there were significantly older oysters (Mann–Whitney *U* test, *P* < 0.01) in the Mesolithic (mean: 4.9 y, median: 3.0 y, maximum: 23 y, n = 923) than the Neolithic (mean: 3.7 y, median: 3.0, maximum: 11 y, n = 501) (Fig. 2*B*). While the periods have differing maximum oyster ages, the minimum oyster ages is always 1 y. The oldest/largest specimens are found in the Mesolithic, with maximum ages ranging from 4 to 23 y, compared to between 5 and 11 y in the Neolithic. When both periods are present at an archaeological site, the Neolithic specimens are consistently smaller. For six of the sites spanning the Mesolithic and Neolithic, we compared the oyster ages between periods (Fig. 2*B*) and found a similar pattern to that of the hinges with three of the six sites having significantly older oysters during the Mesolithic (Mann–Whitney *U* test; Havnø: *P* < 0.01; Eskilsø SØ: *P* < 0.01; Norsminde: *P* < 0.01) and no significant difference at the other sites (Mann–Whitney *U* test; Krabbesholm II: *P* = 0.08; Bjørnsholm: *P* = 0.09; Visborg: *P* = 0.70). The nonsignificant outcomes at those sites could also be a result of the smaller sample size for age determinations compared to size measurements.

### Residuals.

**Fig. 3.**
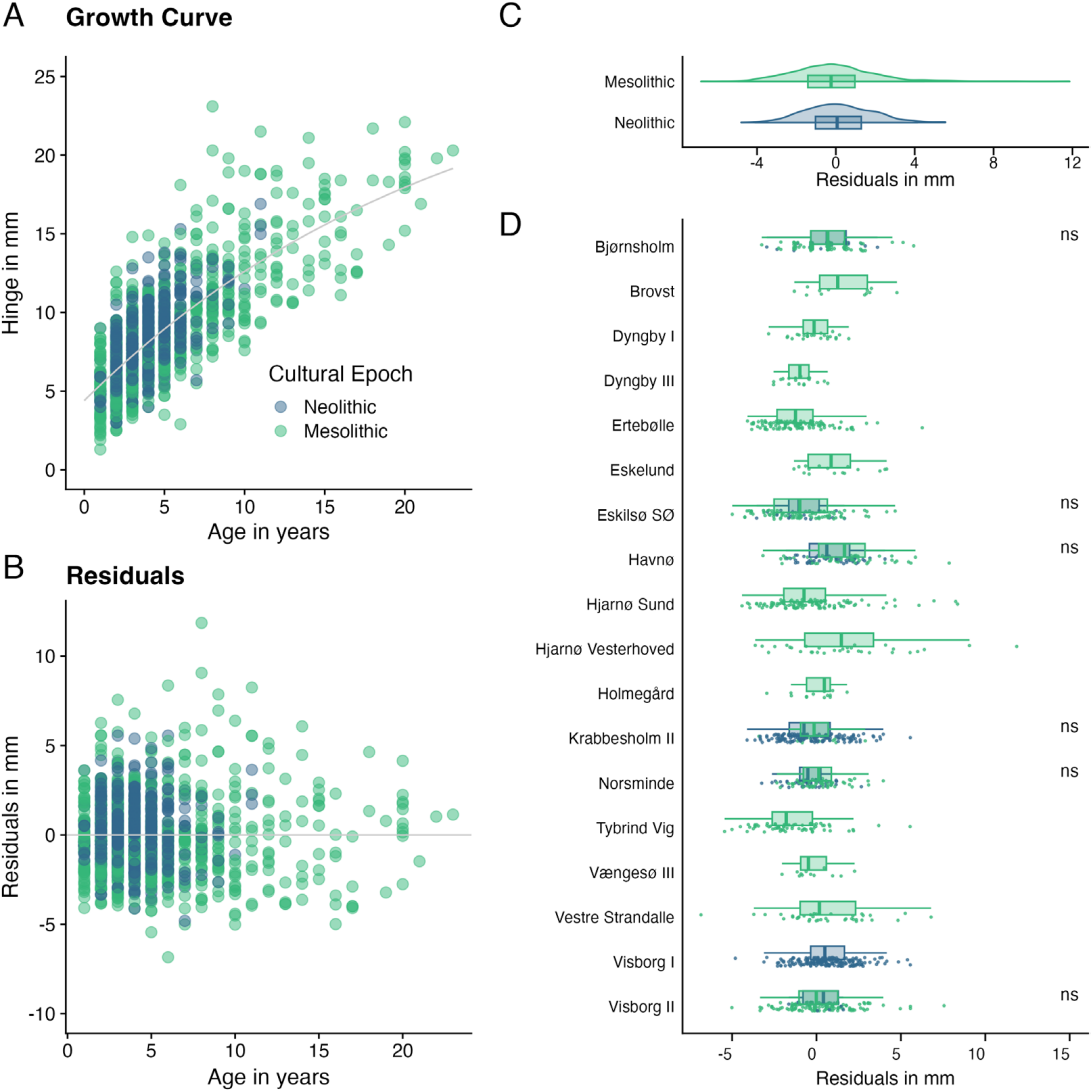
Growth curve (*A*) and residuals (deviations from the growth curve) (*B*) at specific ages. Measured in mm distance from the growth curve at the time of death with negative values indicating smaller than usual shells and positive values indicating larger than usual shells. (*C*) and (*D*) show the residuals in mm from the general growth curve by period and site, respectively, with “ns” indicating no significant differences between Mesolithic and Neolithic distributions following Mann–Whitney *U* tests.

We established a growth curve (*Materials and Methods* and Fig. 3*A*) to better compare differences in growth rates at specific ages. The deviations from the growth curve (Fig. 3*A*) in the form of residuals show whether a shell has grown faster or slower than others of that age (Fig. 3*B*). This was undertaken to remove the impact biological age has on the size of the shell so as to focus on growing conditions alone. Environmental factors of growing conditions, such as water temperature, salinity, and food availability, play a crucial role in influencing oyster growth rates (49, 50). Variations in water temperature can either enhance or impede metabolic processes, directly affecting growth. For instance, warmer temperatures may accelerate growth up to an optimal point, beyond which stress and mortality increase. Salinity levels also significantly impact oyster physiology; oysters thrive in moderate salinity but can experience stunted growth or even mortality in conditions that are too fresh or too saline. Additionally, the abundance and quality of phytoplankton, which constitutes the primary food source for oysters, determine the energy available for growth (51). Periods of low food availability, often due to seasonal changes or environmental disruptions, can lead to slower growth rates. By quantifying growth rates, we thus better understand whether a small shell is simply young when collected or has experienced stunted growth during its lifetime. We compared the residuals across periods (Fig. 3*C*), resulting in significantly faster growing shells (Mann–Whitney *U* test, *P* < 0.01) in the Neolithic (mean: +0.2 mm) than the Mesolithic (mean: –0.1 mm). At the six sites covering both the Mesolithic and Neolithic, we compared the growth rates of both periods and found that none of the sites had significant differences between the Mesolithic and the Neolithic (Fig. 3*D*) (Mann–Whitney *U* test; Krabbesholm II: *P* = 0.51; Bjørnsholm: *P* = 0.89; Visborg: *P* = 0.85; Eskilsø SØ: *P* = 0.98, Havnø: *P* < 0.19; Norsminde: *P* < 0.17).

### Stratigraphic Levels.

**Fig. 4.**
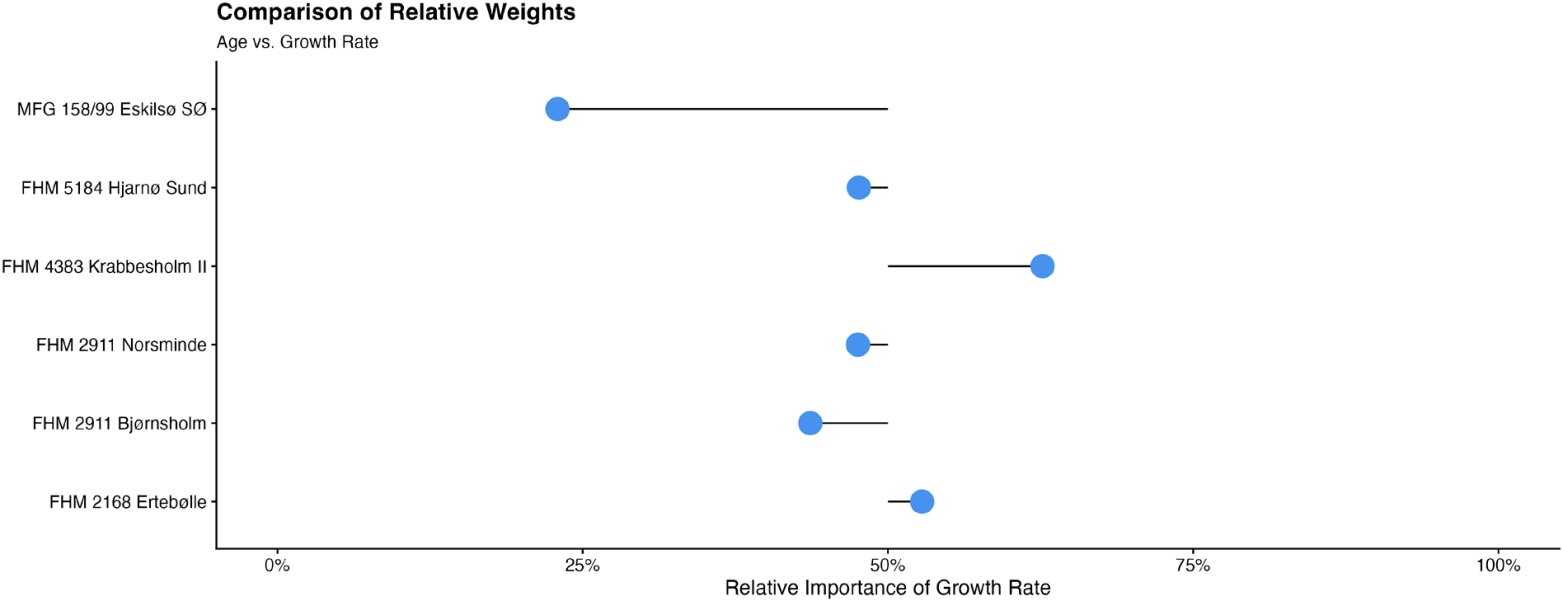
Comparison of relative weights.

The cultural-period scale of analysis was suitable to compare larger trends but does not take into account that many of the sites are not easily comparable because of their different histories of accumulation so that some periods are over- or underrepresented in the dataset. We thus selected sites with more detailed stratigraphic information to better understand the development of oyster sizes across time and — where applicable—across the Mesolithic-Neolithic transition. A relative weight analysis (see Materials and Methods) indicated which factor (age or growth rate) is the controlling factor on the oyster hinge size across the stratigraphy in percent (Fig. 4 and Table 2). This control varied between sites with some being influenced by the growth rate by up to 63% (Krabbesholm II) while others showed only a small influence of the growth rate of 23% (Eskilsø SØ) (all other sites displayed in *SI Appendix*, Figs. S4–S9).

**Table 2.** Results of relative weights analysis with the respective control of shell size

Site	Age	Growth rate
Krabbesholm II	33%	67%
Ertebølle	43%	57%
Norsminde	50%	50%
Hjarnø Sund	53%	47%
Bjørnsholm	56%	44%
Eskilsø SØ	75%	25%

**Fig. 5.**
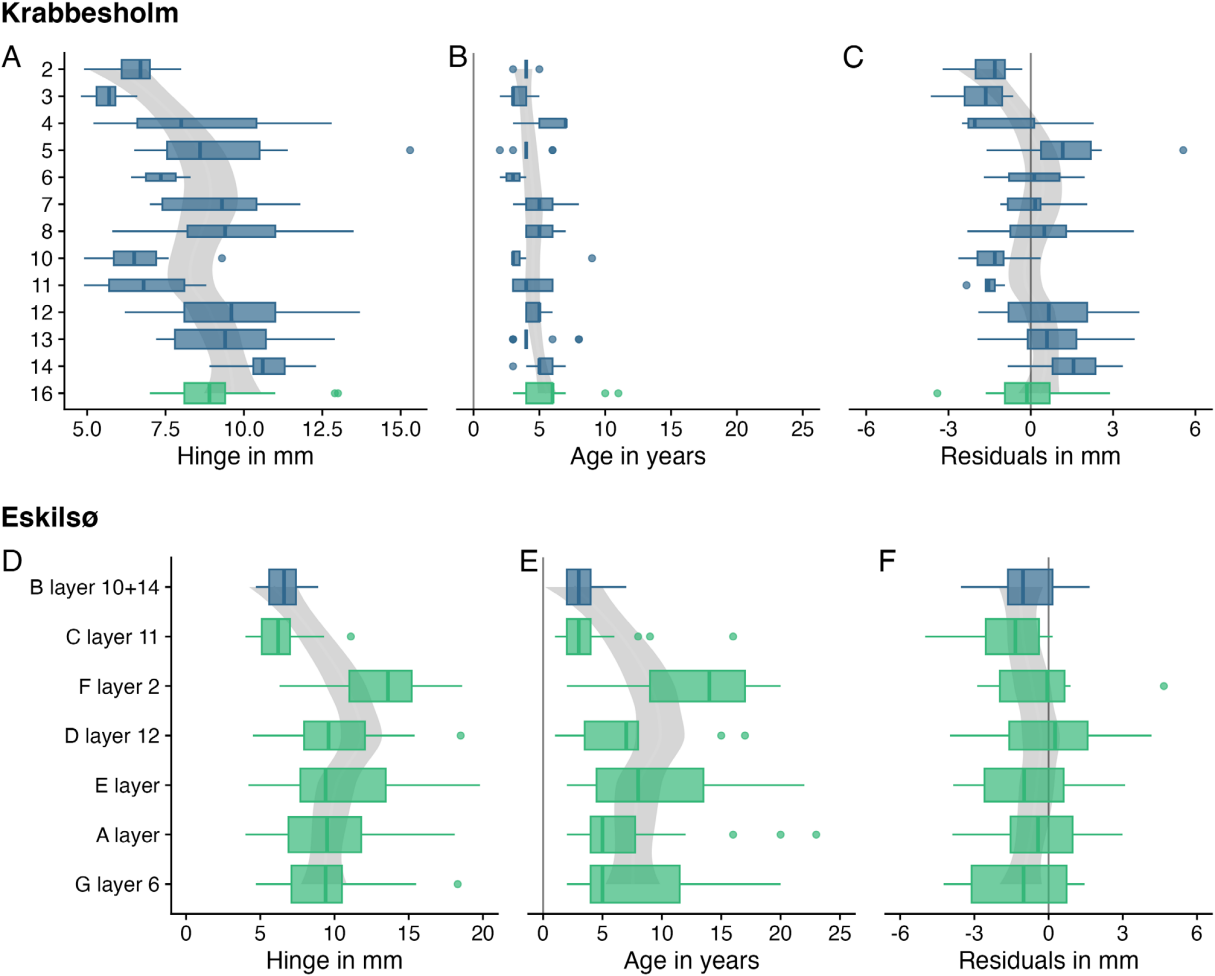
Stratigraphic sequence of hinge, age, and residual data. (*A*–*C*) Krabbesholm II. (*D*–*F*) Eskilsø SØ. Mesolithic and Neolithic layers are shown in green and blue, respectively.

At the Krabbesholm II shell midden only one stratigraphic layer dates to the Mesolithic, making comparisons between the two periods difficult. Despite this limitation, the stratigraphic information allowed us to track temporal variations in oyster size (Fig. 5 *A*–*C*). Interestingly, these changes appear to follow characteristic fluctuations, with oyster sizes decreasing from layers 14 to 10, then increasing from layers 8 to 5, and finally decreasing again from layers 4 to 2. At the same time, the ages of the oysters also exhibit certain increases and decreases; however, the average age remains relatively stable. This contrasts with the interpretation of Milner (46), who found that oyster ages varied in tandem with their size (larger oysters tended to be older), and thus suggested that changes in demographic structure were the main determinant in size. While layer-to-layer changes in age move in tandem with size, these changes are not sufficiently large to explain the entire variability in size. By quantifying the growth rate, which varies strongly across the layer sequence, we show that more pronounced changes of the environment over time seem to be more in control of size than age alone. Contrary to Krabbesholm II, the layers at the Eskilsø SØ shell midden mostly cover the Mesolithic, with only one layer representing the Neolithic period (Fig. 5 *D–F*). One might argue that the chronological difference is why the sites are at the respective opposites of the spectrum regarding the importance of growth rate. However, other sites that are exclusively from the Mesolithic period fall somewhere in between these two, suggesting that the chronological differences between Eskilsø SØ and Krabbesholm II are likely coincidental. At Eskilsø SØ, there is a notable increase in oyster sizes over time (peaking in layer F2) and subsequently diminishing slightly by the end of the deposit (layer B10 + 14), compared to the initial sizes (layer G6). The age data show that oysters were initially consumed at older ages, which aligns with the trend in size data. Over time, especially in the latter stages of the Mesolithic and into the Neolithic, oysters were consumed at younger ages. Throughout this sequence, growth rates exhibit relative stability.

In contrast, the other sites in the study with more detailed chronologies, namely Ertebølle, Hjarnø Sund, Norsminde, and Bjørnsholm, demonstrate a different dynamic wherein neither age nor growth rate is the primary determinant of larger or smaller shell sizes. Specifically, at Ertebølle, Hjarnø Sund, and Norsminde, both age and growth rate undergo simultaneous changes, indicating that both human exploitation and environmental shifts are influencing the oysters. At Norsminde, there is a change in the relative abundance of oysters to cockles (*C. edule*) across the Mesolithic-Neolithic transition. Interestingly, people exploited oysters at a younger age in the Neolithic. This decline does not occur abruptly but is slightly delayed. The growth rates also experience a decline, which is gradual and begins in the Mesolithic. Hjarnø Sund experiences a change in species composition from layers 4 to 5 and above, with an increased proportion of cockles (*C. edule*), as highlighted in (45) and (52). This shift may explain the increased collection age of oysters. However, the reason for this transition to cockles remains ambiguous, especially considering that oysters were experiencing accelerated growth. Finally, the Bjørnsholm shell midden exhibits some fluctuations in the size of the oysters, but more intriguingly, the age and growth rates change in nearly inverse directions. Specifically, ages tend to rise as the Mesolithic-Neolithic transition approaches, while growth rates decline. Growth rates begin to recover in the Neolithic, coinciding with a reduction in the mean age, suggesting an increase in harvesting pressure. We also included the Havnø shell midden in this analysis, which showed a strong influence of the age variable (Age 70%, Residuals 30%). However, due to the short sequence (n = 3) resulting in a weak *p*-value, we removed it from further analysis (see *Materials and Methods*). Havnø does however exhibit a steep decline in oyster size and age, starting with older and larger oysters in the Mesolithic and ending with younger and smaller oysters in the Neolithic.

## Discussion

### Prehistoric Stability in Oyster Harvesting.

Our growth rate analysis of over 2,000 European oyster (*O. edulis*) shells reveals that oyster harvesting stayed for the most part within sustainable margins across the recorded ~3,000 y time period. At almost all sites (excluding Eskilsø SØ), neither evidence of intensified human harvesting pressure nor a decline in growth rates are such as to indicate that oyster populations were at imminent risk of collapse or abandonment as a food resource between ~5,660 to 2,600 cal BCE. That said, oysters had become quite small and were only 2 to 3 y old when harvested, demonstrating that the oyster population was under somewhat greater harvesting pressure or at least the nearshore part of the population that was accessed by people. Our quantification of the two factors influencing oyster size (harvesting pressure and growth rate) revealed that at most sites both are equally at play (Table 2). This means that environmental impacts on growth rate are not to be excluded but that the human impact is strong enough to influence the demography of the local oyster population (see also ref. 34). This lends further credibility to the usefulness of size-data alone as a viable proxy for demographic change, without taking into account individual ages, as has been employed elsewhere on a larger scale (31, 32). One example of the impact that human harvest can have during stable growing conditions is provided by the Eskilsø SØ shell midden. Here, the data show a steep drop in oyster size and age during the Mesolithic with no sign of recovery at the start of the Neolithic and no changes in growth rate that could explain the drop in size. This is the only site where a decline in oyster size predates the oyster-to-cockle shift and can be explained by human impact. In this case, the evidence is consistent with the hypothesis that the shift to *C. edule* occurred because of overexploitation of the oysters. At each of the other sampled sites harvesting pressure fluctuates but generally recovers, suggesting that overexploitation of oysters was not a factor in the change of dominant species. Similarly, cockles might have become more abundant because of environmental changes favorable to them, but without any change in the oyster demography or size. Also, people might have exploited cockles more intensively than before because of changes in economic scheduling associated with the agricultural calendar.

### Abandonment of Oysters.

Against our hypothesis of an expected age increase in European oysters (*O. edulis*) with the onset of agriculture, our dataset reveals no such trend in Denmark. Instead, oyster ages and sizes decrease from the earliest part of the Ertebølle culture and become stable throughout most of the period with little changes linked to each site’s transition to the Neolithic (46). The lack of a connection between oyster size changes and the Neolithic transition agrees with recent dating of the oyster-to-cockle shift at the submerged site of Hjarnø Sund. Here, the shift occurs between ~5,500 to 5,300 and ~5,300 to 5,200 cal BCE, which is around the time of the transition from the Middle Mesolithic Kongemose to the Late Mesolithic Ertebølle and around 1,300 y prior to the Mesolithic-Neolithic transition (45). This earlier occurrence points toward local environmental changes as the main cause, creating nearshore habitats more suitable for *C. edule* (and *M. edulis*) than for oysters and thus reducing the overall availability of oysters without impacting their growth rates. The sudden change of this transition suggests a tipping-point scenario, such as the one described by Larsen et al. (45), where sea-level-related geomorphological developments of beach ridges and increased sedimentation removed the potential for young oysters to attach themselves and grow to a size worth harvesting. Such a scenario is similar to the fate of a recent *O. edulis* bank off Helgoland, northern Germany, which struggled under increased sedimentation (53). Environmental changes such as these interrupt or rather prevent the shell growth we identified in this study, therefore their impacts happen so quickly that they cannot be identified with the sclerochronological methods used in this study. Interestingly, the shift to another mollusk species seems to have happened effortlessly and it is not entirely unlikely that a shift in harvesting practice and the local communities’ adaptability had a big role to play in the swiftness of the transition.

### Late Mesolithic Sedentism.

Throughout Europe, the Ertebølle culture provides an outstanding opportunity for understanding the lifeways of hunter-gatherer-fisher communities during the Mid-Holocene due to the density of sites and richness of the record. It is generally assumed that these communities were seasonally mobile, frequently returning to the same locations but without necessarily establishing permanent settlements, unlike the sedentary agricultural communities of the Neolithic. One might argue that mobile communities would not provide an ideal case study for examining the sustainability of long-term *O. edulis* harvesting, as it is difficult to capture their spatially extensive footprint. In fact, in our dataset, size and age ranges of oyster shells at Mesolithic sites were much larger than those of the Neolithic. But these wide ranges are not representative of all Mesolithic sites. Indeed, there are many sites that have just as small as, or even smaller mean sizes of oyster shells than those from the Neolithic. The sites that do contain large shells are either the earlier shell midden sites (e.g., Hjarnø Sund, Hjarnø Vesterhoved), or sites that may have been visited less often than their regional counterparts which were more centrally accessible (i.e., located on an island, for instance, Eskilsø SØ, Havnø, Hjarnø Sund, and Hjarnø Vesterhoved).

At Hjarnø Sund, the earliest layers date to the Kongemose–Ertebølle period and indicate larger sizes which decrease during the Ertebølle proper and increase again afterward, potentially pointing to shifts in local resource use or changing mobility. In comparison, oysters at Brovst dating to the mid-5th millennium cal BCE (i.e., Layer 4), have similarly young ages to those from other later Ertebølle sites. Taken together, these data indicate that harvesting pressure was consistently high for around the first ~1,000 y of the Ertebølle period, indicating that semisedentary mobility could have been practiced. Thus, arguments based on assumptions of reduced harvesting pressure in this early period because of smaller or more mobile human groups cannot apply here. Havnø, which is situated in the Mariager Fjord and around 2.5 kilometers from the vast Visborg shell midden, with which it shares similar growth rates, is an example for a later but perhaps less frequently visited site, because the Mesolithic layers of Havnø have much larger and older *O. edulis* than at Visborg. One explanation is that during the earliest occupation at the Havnø shell midden, a pristine or underused oyster bed was present near to the site (46), which experienced less harvesting pressure as it was visited less frequently than others, where average ages dropped faster. Similarly, large mean ages and sizes were evident during the Mesolithic at the Eskilsø SØ shell midden. The site is also situated on an island in Roskilde Fjord, while more central contemporaneous sites were located along the mainland (54). Since they might not have been visited more frequently, the oyster bed near Eskilsø SØ would have been less affected, allowing oysters to grow older between harvests. In general, older oysters are predominantly found in layers spanning the transition from the Middle to Late Mesolithic (Kongemose-Ertebølle periods), i.e., relatively early in the Mesolithic sequence, or at locations that are more remote. In comparison, the oyster ages from the majority of the Ertebølle layers, which are later in date, are younger and, in fact, comparable to the Funnel Beaker oysters. This similarity points toward a consistent harvesting pressure across the Mesolithic-Neolithic transition. The pressure that local Ertebølle communities exerted on the oyster beds adds to the growing evidence that a limited mobility pattern similar to Neolithic settlers seems more likely than a traditional Mesolithic mobility system that operates on a seasonal basis.

These data align with evidence elsewhere for a sedentary lifestyle and subsistence economy, for instance, the Early Mesolithic site of Norje Sunnansund, Sweden (55). Here, the constant and predictable fish supply, coupled with intensive, year-round fishing activities, contributed to a sedentary lifestyle. Given the sedentary lifestyles, processes for regulating fishing management and potentially fishing/shellfishing rights could be inferred for the Ertebølle period. Some degree of territoriality has been discussed and goes hand in hand with the evidence of increased trauma on human skeletal remains (56, 57), potentially linked to competition over access to prime fishing locations (58). Given the need for labor coordination to build and maintain stationary fishing structures, the control over these areas likely necessitated some level of structured social organization. Continuous presence at some of the coastal settlements, particularly those with evidence of multiple seasons of occupation, argues for a quasi-managerial function that may have been implemented to oversee fishing activities and resource distribution. It is plausible that these developments in resource management, in combination with the need to safeguard coveted fishing grounds, contributed to a heightened sense of territoriality. Thompson et al. (32) suggest that individual and local resource management practices would have contributed to long-term sustainability on a regional scale, similar to the sustainable use of oysters that is found in our dataset.

### Implications for Future Marine Ecologies.

Anthropogenic pressures in the industrial period have led to a significant decline in the populations of Ostreidae. In response, several repopulation initiatives across Europe and beyond have emerged (12, 21). These initiatives focus on aquaculture techniques, disease management, and habitat restoration (59). The goal of implementing these strategies is not only to reestablish the ecological role of Ostreidae but also to rejuvenate the fisheries. As mentioned above, oysters act as biofilters, habitat providers, and food sources for various marine species. Their restoration can therefore significantly contribute to the health of marine ecosystems, with benefits extending to other marine resources and overall food security. NORA (Native Oyster Restoration Alliance) in particular, has undertaken various initiatives across Europe, including the UK and the Baltic Sea region (1), as have others on the western side of the Atlantic (19–21). These initiatives encompass breeding programs, oyster bed restoration projects, and disease management strategies, seeking to rebuild self-sustaining oyster populations across their former ranges. These initiatives underscore the importance of a multidisciplinary approach that combines ecological, social, and economic aspects to restore populations effectively. It is crucial to continue such endeavors to halt and reverse the decline of populations in these historically important regions, ensuring the sustainability of these ecosystems and the industries they support. With current oyster populations being in a diminished state and many previously rich environments having been removed during the last 200 y, no substantial long-term perspective exists. While long-term ecological studies of the North Sea are looking to the past, they rarely go beyond the last 200 y. A lack of substantial material of oyster harvests from before the period of peak exploitation during the 1800s, is thus a large gap in our understanding of long-term resilience of these oyster beds.

The data presented here extend the historical record back to ~7,660 y ago and span a period of more than three millennia of coastal activity. The metrics derived from these data, indicating robust patterns of harvesting methods and potential reef management strategies, significantly enhance our understanding of oyster use in the prehistoric past. Without incorporating these datasets, gaining insights into the resilience of long-term harvesting practices would have been unachievable. As such, they provide a helpful reference to modern studies of oyster populations (60–62). Importantly, the use of prehistoric ecological data affords practical insights and metrics for restoration projects. These include selecting suitable locations, understanding the productivity of these areas in the past, and determining their capacity to support human activities (63). By calculating growth rates, we can establish expectations for the annual growth of restored modern populations, providing an empirical baseline for comparison and a target to work toward. This enables more reliable planning and forecasting for the duration of restoration efforts. Of additional importance are the comparatively large and old shells. These do not occur throughout the studied period but are most commonly collected during the Mesolithic with many of them dating to the early Ertebølle culture. Stone Age oyster gathering appears to have been usually carried out in the nearshore sections of reefs, but occasional lower-shore gathering or initial collections can provide a glimpse into the rest of the lower-lying and less harvested population, which acts as the broodstock for the nearshore specimens. The larger shells found there, and in initial collections of early Ertebølle layers, can thus be used as a guide of what the critical broodstock of a sustainably but consistently harvested reef section looks like (i.e., up to 124 mm in length, up to 23 y old and with growth rates of 100 mm within 6 y) (*SI Appendix*, Fig. S12). Oyster fisheries, and potentially other fisheries, could use metrics such as these (i.e., parameters of top shells) as one indicator to determine a) whether they are harvesting a healthy reef; b) whether their repopulation efforts have reached sufficient ecological levels; and (c) to protect those parts of a reef where these fecund oysters occur, which are the most important for the reproduction of oysters in the wider area. In addition, the low minimum ages of 1 y found in most sites suggest that there was little active exclusion of younger specimens, possibly because the reproductive capacity of the reef was already sufficient without needing to preserve younger oysters for population sustainability. Finally, this detailed examination of sustainable oyster harvests underscores the deep connections between coastal communities and oysters, further substantiating and legitimizing the efforts put forth by conservation projects. It also underlines the risk of losing invaluable paleoecological records due to coastal erosion and rising sea levels. Moreover, it draws attention to the past destruction of shell midden deposits elsewhere in Europe and beyond, which have been exploited for agricultural purposes and are now predominantly found in damaged conditions, with many examples in Denmark, northern Germany, and the United States. This loss of ecological archives poses a serious challenge for researchers and underscores the importance of protecting these sites.

## Conclusion

Our study reveals the intricate relationship between *O. edulis* age, environmental factors, and the sustainability of oyster harvesting practices. These findings contribute to our understanding of the historical dynamics of the Danish oyster population and can inform conservation strategies for maintaining healthy oyster beds in the future. We found compelling evidence of the long-term stability of sustainable oyster harvesting practices at almost all sites. Our findings also revealed that, on average, age accounted for approximately half of the variability in oyster size. Surprisingly, we did not observe a visible change in oyster growth rates prior to or during the oyster-to-cockle shift. However, we did identify a size shift potentially related to harvesting pressure in the earliest Ertebølle layers, from which the oyster population did not subsequently recover. We argue that this persistent pressure provides broad evidence for sedentary behavior and territoriality during the Late Mesolithic period. Most importantly, this work highlights the importance of growth rates in preindustrial datasets that can inform research regarding the long-term stability and sustainability of mollusk harvesting.

## Materials and Methods

### Materials.

The results obtained from 529 European oyster (*O. edulis*) samples are presented here, complementing “legacy data” on 1578 *O. edulis* samples (46, 48, 64–66), which was reanalyzed (54, 67). The overall dataset represents 2107 oysters from 20 archaeological sites, primarily shell middens, but also inclusive of coastal sites with cultural and/or natural layers containing shells such as FHM 3954 Dyngby I and FHM 4339 Dyngby III, and the natural shell bank at the submerged site of FHM 2033 Tybrind Vig, located across Denmark (Fig. 1, Key reference(s) for site in *SI Appendix*, Table S1). Based on radiocarbon (^14^C) dates measured on a range of artifacts and ecofacts, including human and faunal remains, charcoal, organic residues adhering to pottery, mollusks, wood and bark, and typo-chronologies of material culture (e.g., lithics and the presence/absence of ceramics), the sites date from the mid-late 6th to the beginning of the 3rd millennium cal BCE (Key reference(s) for site in *SI Appendix*, Table S1). Similarly, the shell samples were assigned to the Ertebølle and/or Funnel Beaker cultures via association with directly dated materials as well as typo-chronologies of material culture, shell midden matrices, and other forms (presence/absence) of material culture.

The majority of the oyster samples were taken when the excavations were being conducted and differed on a site-by-site basis. For instance, the column samples, which enabled us to compare size, age, and growth rates stratigraphically in this study, were directly excavated by H.K.R. and N.H. (ÅHM 6814 Visborg) as well as N.M. (FHM 4383 Krabbesholm II) using hand tools (i.e., brushes, trowels, shovels, dustpans, buckets, spades, wheelbarrows, etc.). The four columns from ÅHM 6814 Visborg were cut directly into the “cleaned” sections (i.e., ~2 to 3 cm of the shell deposits were excavated prior to sampling) where the thickest shell deposits were encountered to gain an impression into how the shell deposits built up, to determine where the prehistoric shoreline was located and to assess seasonality. Columns 1 to 3 measured 50 × 50 cm, while column 4 measured 25 × 25 cm. They were excavated following 10 cm arbitrary spits as opposed to layers (see ref. 68). Columns 1 to 3 were excavated to obtain oyster shells every 10 cm for sclerochronology. Column 4 was primarily excavated to obtain bulk sediment samples for macrofossil/phytolith analysis. Although this resulted in differences in the number of oyster samples per column available for analysis (Column 1, n = 49; Column 2, n = 59; Column 3, n = 64; Column 4, n = 6), we focus primarily on the data obtained from Columns 1 to 3 given their broadly similar sample sizes. The remaining oyster samples from ÅHM 6814 Visborg were taken from across the shell midden during the 2017 and 2018 excavations. They were initially intended for radiocarbon dating which did not take place. The column from FHM 4383 Krabbesholm II was excavated through the shell midden sequence for the sole purpose of a detailed seasonality study. Column 7737, which measured 50 x 50 cm, was excavated by context (i.e., through 17 layers) which was based on changes in the shell matrix (i.e., mollusk content, color, soil consistency, and inclusions). Oyster samples were extracted from each layer during the 2004 excavation for sclerochronology (46, 65, 69). “Legacy” column samples were also available (FHM 2168 Ertebølle, and FHM 2911 Norsminde). The J-column from FHM 2168 Ertebølle was initially taken for an in-depth shellfish study (70). It measured 20 x 20 x 186 cm and was divided into 26 layers, of which 16 were sampled. S.H.A. and N.M. collected oyster samples from the archives at Moesgaard Museum in 2010. Column N77 from FHM 2911 Norsminde was similarly removed for detailed analysis and measured 100 x 100 cm. It was excavated in 1977 in arbitrary 10 cm spits, following a chronological sequence, and had been sampled previously by N.M. (48, 69). For further details concerning the sampling, see refs. 48, 67, 69.

The remaining shell samples were collected either when the excavations were being conducted or from museum archives by us: H.K.R. (VMÅ 2185 Ertebølle, FHM 4014 Havnø, FHM 2033 Tybrind Vig and ÅHM 6814 Visborg), P.M.A. (FHM 5184 Hjarnø Sund, FHM 5948 Hjarnø Vesterhoved and FHM 6218 Vestre Strandallé), S.A.S. (MFG 158/99 Eskilsø SØ), S.H.A. (FHM 2911 Bjørnsholm, FHM 1586 Brovst, FHM 3954 Dyngby I, FHM 2168 Ertebølle, FHM 1116 Eskelund, FHM 4014 Havnø, FHM 1532 Holmegård, FHM 2033 Tybrind Vig and FHM 4428 Vængesø III), and N.M. (FHM 3954 Dyngby I, FHM 4339 Dyngby III, FHM 4383 Krabbesholm II, FHM 2033 Tybrind Vig and FHM 3933 Visborg). In the following, we will briefly summarize the oyster samples that are presented here.

We targeted oyster samples from some of the latest Kongemose/earliest Ertebølle sites in Denmark (e.g., FHM 5184 Hjarnø Sund and FHM 5948 Hjarnø Vesterhoved). The oyster samples from FHM 5184 Hjarnø Sund are derived from Layers K19 (oyster shell layer) and/or K21 (cockle shell layer) of shell heap 2 which was excavated in 2015, while those from FHM 5948 Hjarnø Vesterhoved were taken from Layer L11 (mollusk shell layer) that was excavated in 2018. Excavations at both sites were led by P.M.A. and were undertaken using a diver-operated ejector pump in 25 cm increments (see refs. 45, 52, 71, 72). Furthermore, we sampled opportunistically such as at the site of VMÅ 2815 Ertebølle, near to the eponymous shell midden in the Limfjord, while infrastructure-based development (expansion of the Aarhus Light Rail link) enabled us to sample a previously unknown shell midden in the Bay of Aarhus (FHM 6218 Vestre Strandallé). The oyster samples from VMÅ 2815 Ertebølle were located directly above the glacial till, and were taken by H.K.R. during a site visit with S.H.A. to a small-scale rescue excavation by Vesthimmerlands Museum in 2015. In contrast, those from FHM 6218 Vestre Strandallé are derived from Layer 7 and were taken directly from the section by P.M.A. during excavations carried out by Moesgaard Museum in 2020. Finally, we included oyster samples from the natural shell bank at the submerged site of FHM 2033 Tybrind Vig which were collected from the archives at Moesgaard Museum by S.H.A. and N.M. in 2010 and S.H.A. and H.K.R. in 2015 (see refs. 67, 73, 74). For further details concerning the sampling, see refs. 48, 67, 69.

In most cases, oysters were selected based on their potential for clear incremental records once sectioned. This introduces a bias toward shells that are large enough to indicate healthy growth (i.e., +3 mm) but not so large that they might be a very old specimen (i.e., +15 y) with drastically reduced growth rates. That said, the degree of completeness was most often the deciding parameter in sample selection.

### Hinge Measurements.

The hinge of each oyster was measured with electronic calipers in mm to one decimal place (*SI Appendix*, Fig. S3) (67). In total, 1964 measurements were obtained (*SI Appendix*, Fig. S2*B* and *SI Appendix*, Table S1).

### Incremental Growth Line Analysis for Obtaining Age.

A total of 2107 oyster shells were thin sectioned following slight modification (see refs. 67, 75) of the method set out by Milner (36). Briefly, oysters were cut from the tip of the hinge at a right angle to the growth lines using a Buehler ISOMET 1000 Precision Saw (Model 11-2180), and a Buehler Diamond Wafering Blade (Series 15LC Diamond No 11-4276). Then, the samples were embedded in resin (Buehler Epo-Thin Low Viscosity Epoxy Resin No 20-8140-12B and Buehler Epo-Thin Low Viscosity Epoxy Hardener No 20-8142-016), and vacuum impregnated using Buehler Vacuum Impregnation Equipment (Model No 20-1384-220). Once set, the samples were lightly ground using a range of metallographic grit papers (P600, P1200, and P2500 grades, respectively) using a Buehler Motopol 2000 Grinder/Polisher, and polished using a Texmet polishing cloth and Buehler MetaDi 3 μm water based diamond paste. They were bonded to a glass slide using Loctite 322 Adhesive, and then the body of the “resin block” was sliced from the slide using a Buehler ISOMET 1000 Precision Saw, leaving a “thin section” of the resin block and shell of ~50 to 100 μm in thickness. The thin sections were lightly ground by the methods described above, until a thickness of ~10 to 25 μm was achieved.

### Examining the Thin Sections.

The thin sections were examined under polarized light at magnifications of x10 to x40, and the ages of the oysters were determined by counting the annual lines (see ref. 48 and *SI Appendix*, Fig. S4).

### Hinge-to-size Conversion.

We reconstructed the size of *O. edulis* using measurements of oyster shell hinges and total shell lengths from the archaeological sites. Oyster hinge size is a useful proxy value for total size (48) and allows for specimens that are not preserved in their entirety to be included in the analysis because hinges are usually better preserved due to their denser structure. However, we estimated the total length (*SI Appendix*, Fig. S5), which was based on empirical information in Milner (48), resulting in the equation of

length = 35.4 ∗ log(hinge),

with measurements for hinge and length both in mm.

### Estimating Growth Rates.

Oyster growth rates were compared by defining standard sizes for each age and then measuring the shells’ individual deviation from that size in the form of residuals. Hinge size and age were determined using the sclerochronological methods described above and provided the base for a standardized growth curve (Fig. 3*A*). To establish a standardized growth curve for oysters, we employed the von Bertalanffy growth function (76), which is widely used to model the growth of various marine organisms. Our growth function is defined as

$$H_{s}(A)=H_{\infty }(1-e^{-k(A-t_{0})}),$$

where *H_s_(A)* is the predicted shell hinge size at age *A*, *H_∞_* represents the asymptotic shell hinge size the oysters would reach if they grew indefinitely, *k* is the growth rate coefficient, and *t_0_* is the hypothetical age at which the hinge size would be zero. The parameters of the growth function were estimated using the fishmethods package in *R* (77), which implements a nonlinear least squares approach to fit a growth model to our dataset of shell hinge size and corresponding ages of oysters. The objective of the fitting process is to minimize the sum of squared residuals between the observed and predicted sizes:

$$\underset{H_{\infty },k,t_{0}}{\text{min}}\sum_{i=1}^{n}(H_{i}-H_{s}(A_{i}){)}^{2}.$$

This procedure yields the estimated parameters for the standardized growth curve, which provides the baseline for our comparisons of individual hinge growth rates. Following the establishment of the standardized growth curve, individual oyster growth rates were compared by calculating the residuals for each oyster, which represent the deviation of the actual hinge size from the standard size predicted by the growth function. The residual for the *i*-th oyster is defined as

$$r_{i}=H_{i}-H_{s}(A_{i}),$$

where *H_i_* is the observed hinge size of the i-th oyster, and *H_s_(A_i_)* is the standard hinge size for its age according to the growth function. The residual values were then used to compare between specimens and across layers or sites. This approach allowed us to better compare shells’ growth rates between different ages, since shell growth is not linear.

Statistical analysis was performed using *R*, the code is published via an Open Science Framework repository (47) and makes use of specifically multiple regression analysis and relative weight analysis. *P*-values obtained from comparative tests between periods and layers were adjusted for multiple comparisons using the Bonferroni correction.

Multiple regression analysis and relative weight analysis was carried out to better evaluate the influence of age and growth rate. The multiple regression analysis included the use of standardized coefficients and indicated sites with too short sequences to provide reliable samples sizes (i.e., Havnø and the four column samples at Visborg). We also carried out tests for Multicollinearity using Variance Inflation Factors (VIFs), which showed minimal inflation for all factors.

## Supplementary Material

Appendix 01 (PDF)

## Data Availability

*R* code data have been deposited in Open Science Framework repository (http://dx.doi.org/10.17605/OSF.IO/E76WN) (47). All study data are included in the article and/or *SI Appendix*.
