# Supplementary material for: The effects of Mid-Holocene foragers on the European oyster in Denmark
Source: Proc Natl Acad Sci U S A. 2024 Oct 28;121(46):e2410335121. doi: 10.1073/pnas.2410335121 (PMC11573498; doi:10.1073/pnas.2410335121)
Supplement: Supplementary file 1 — Appendix 01 (PDF) [file pnas.2410335121.sapp.pdf]

PNAS

Supplementary Information for

The effects of Mid-Holocene foragers on the European oyster in Denmark

Harry K. Robson[1*], Niklas Hausmann[1,2], Eva M. Laurie[1,†], Peter Moe Astrup[3], Karen Povlsen[4], Søren A. Sørensen[5], Søren H. Andersen[3] and Nicky Milner[1]

[1]Department of Archaeology, University of York, Wentworth Way, Heslington, York, YO10 5DD, United Kingdom

[2]Leibniz Zentrum für Archäologie, Ludwig-Lindenschmit-Forum 1, 55116 Mainz, Germany

[3]Moesgaard Museum, Moesgaard Alle 15, 8270, Højbjerg, Denmark

[4]The Historical Museum of Northern Jutland, Algade 48, Aalborg, Denmark

[5]Roskilde Museum, Sankt Ols Stræde 3, 4000 Roskilde, Denmark

These authors contributed equally: Harry K. Robson, Niklas Hausmann

[+]Corresponding author: Harry K. Robson (harry.robson@york.ac.uk)

[†]Deceased 13th July 2022

This PDF file includes:

Figures S1 to S12

Table S1

SI References

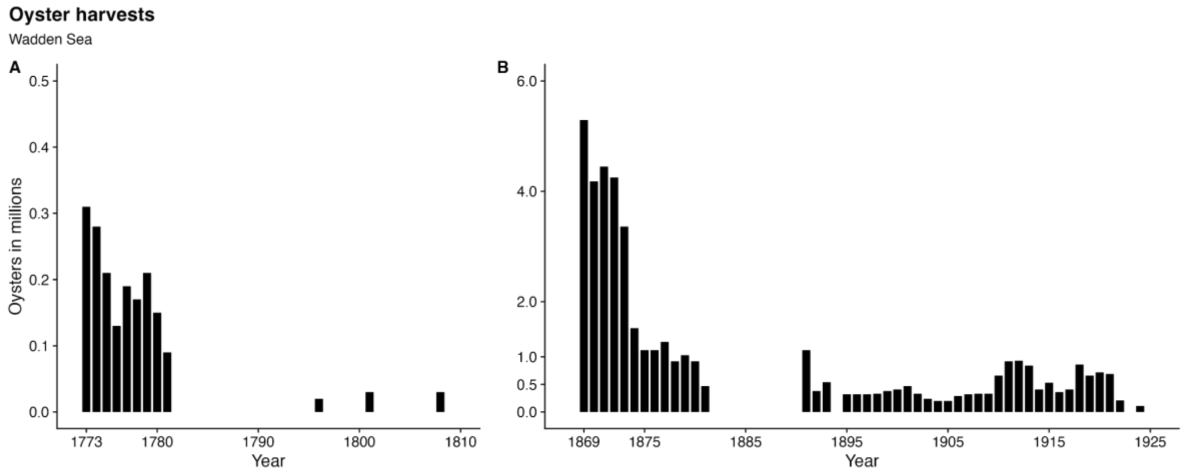

Fig. S1. Oyster harvests in the Wadden Sea. Note the changing y-axis between the two periods of 1773–1810 (A) and 1869–1925 (B) (after Lotze (1)).

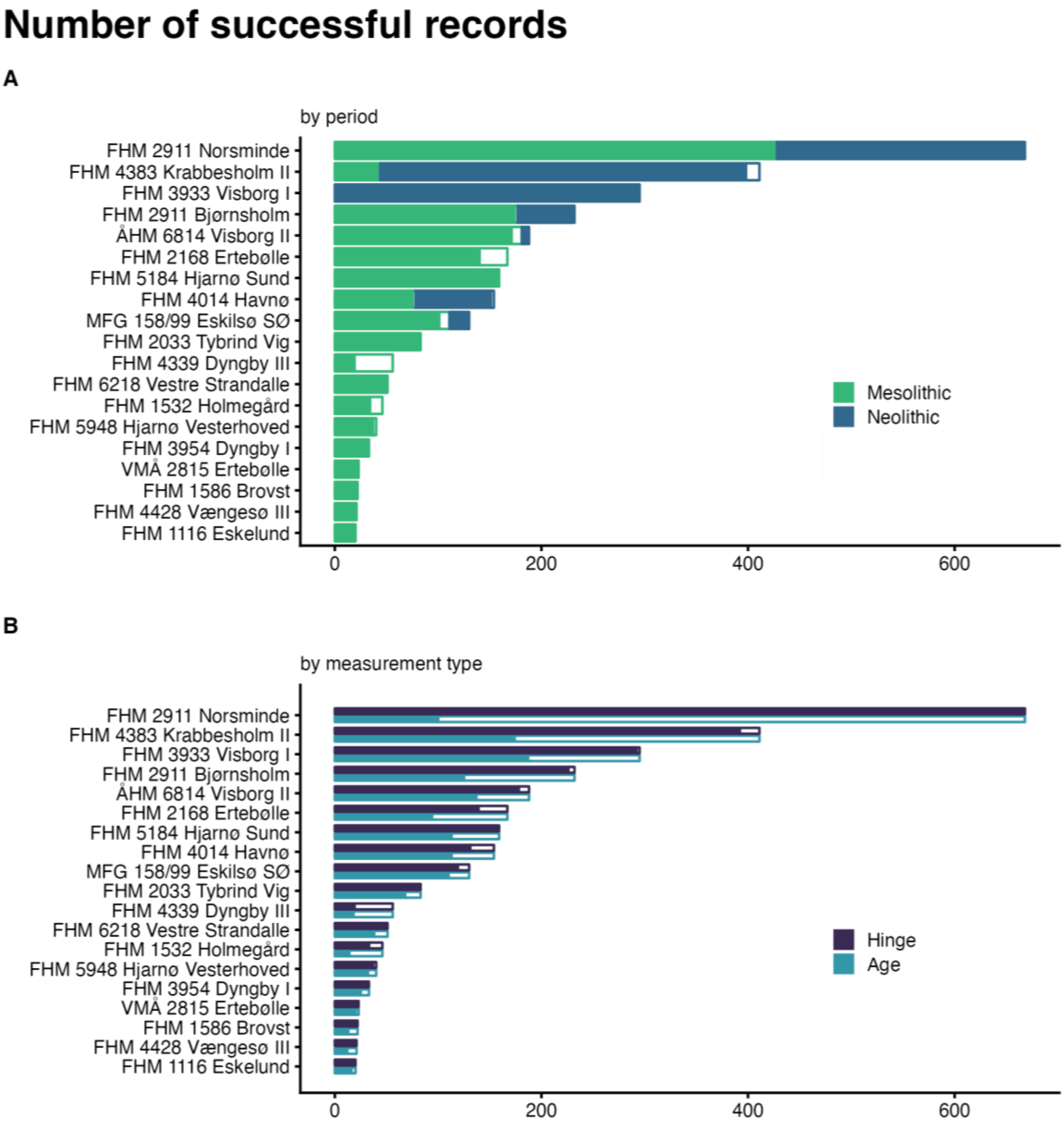

Fig. S2. Sample quantities for the archaeological sites by period (A) and measurement type (B).

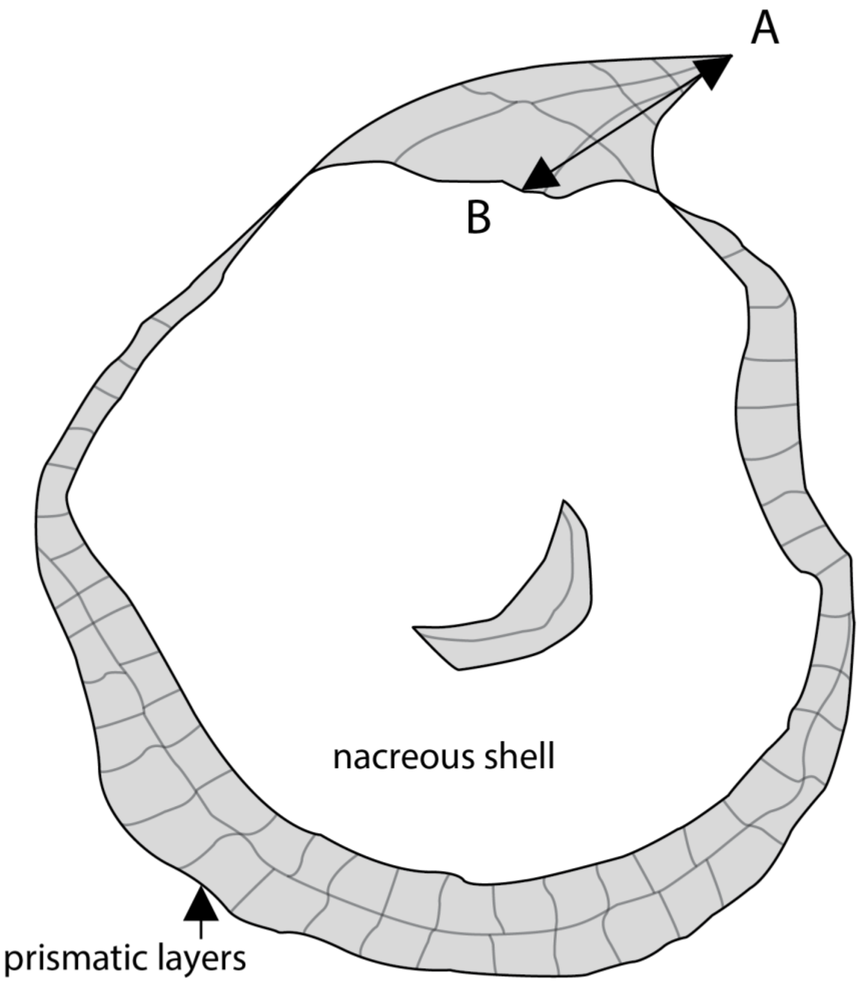

Fig. S3. Diagram showing how the hinge measurements were taken. A–B: measurement along the growing plane (after Milner (2: Figure 3.4).

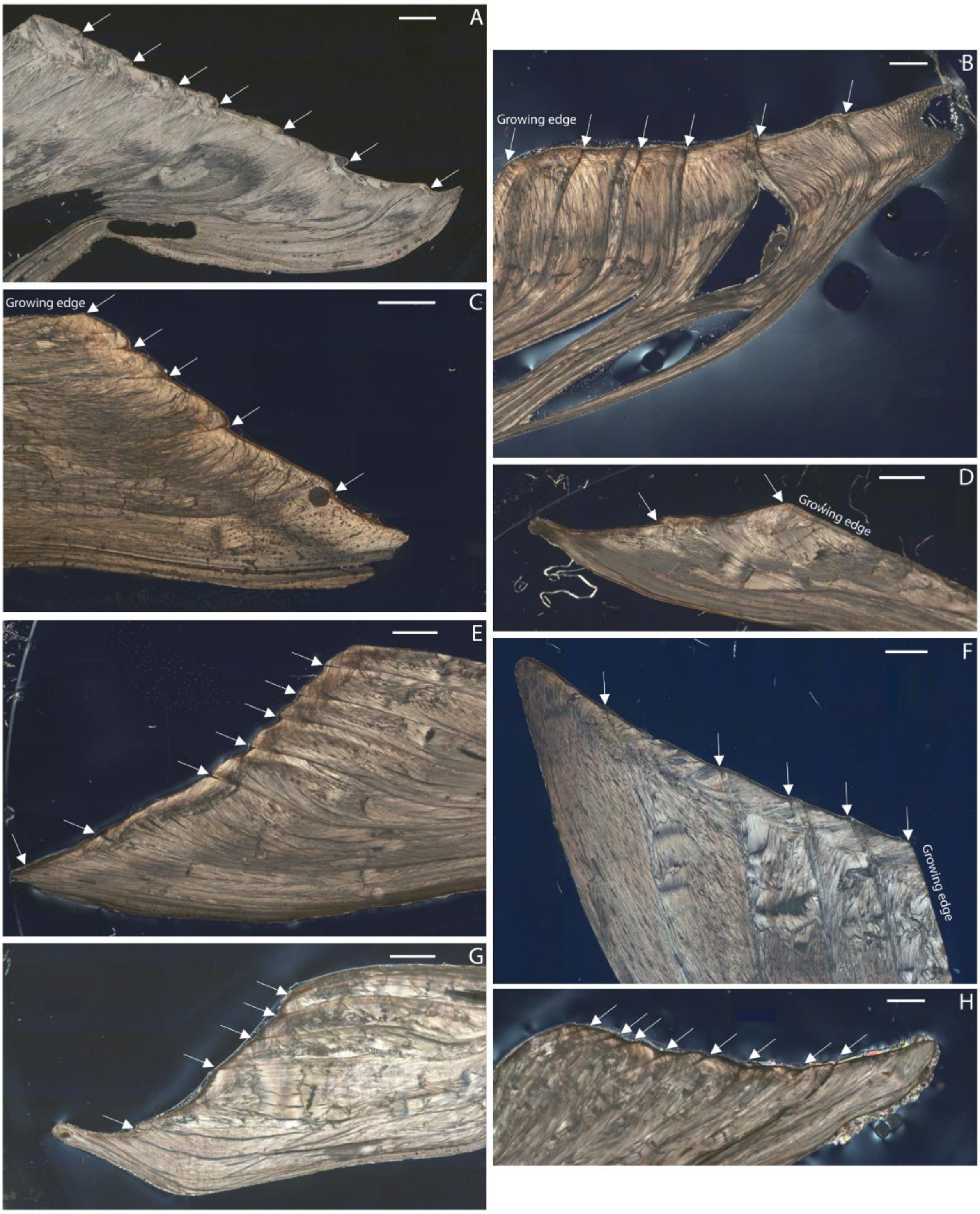

Fig. S4. A selection of thin sections. A: B8.2, an Ertebølle oyster from FHM 2911 Bjørnsholm with seven annual lines. B: J24.16, an Ertebølle oyster from FHM 2168 Ertebølle (*locus classicus*) with a line along the growing edge, and another five annual lines. C: J26.1, an Ertebølle oyster from FHM 2168 Ertebølle (*locus classicus*) with a line along the growing edge, and another four annual lines. D: H.POL.7, a Funnel Beaker oyster from FHM 4014 Havnø with a line along the growing edge, and another annual line. E: H.UED.1, a Funnel Beaker oyster from FHM 4014 Havnø with seven annual lines. F: H.UED.6, a Funnel Beaker oyster from FHM 4014 Havnø with a line along the growing edge, and another four annual lines. G: TV.5, an Ertebølle oyster from FHM 2033 Tybrind Vig with five annual lines. H: TV.6, an Ertebølle oyster from FHM 2033 Tybrind Vig with at least eight annual lines. Note the mineral accretions, displaying third order interference colours. Scale bars: 1000 µm.

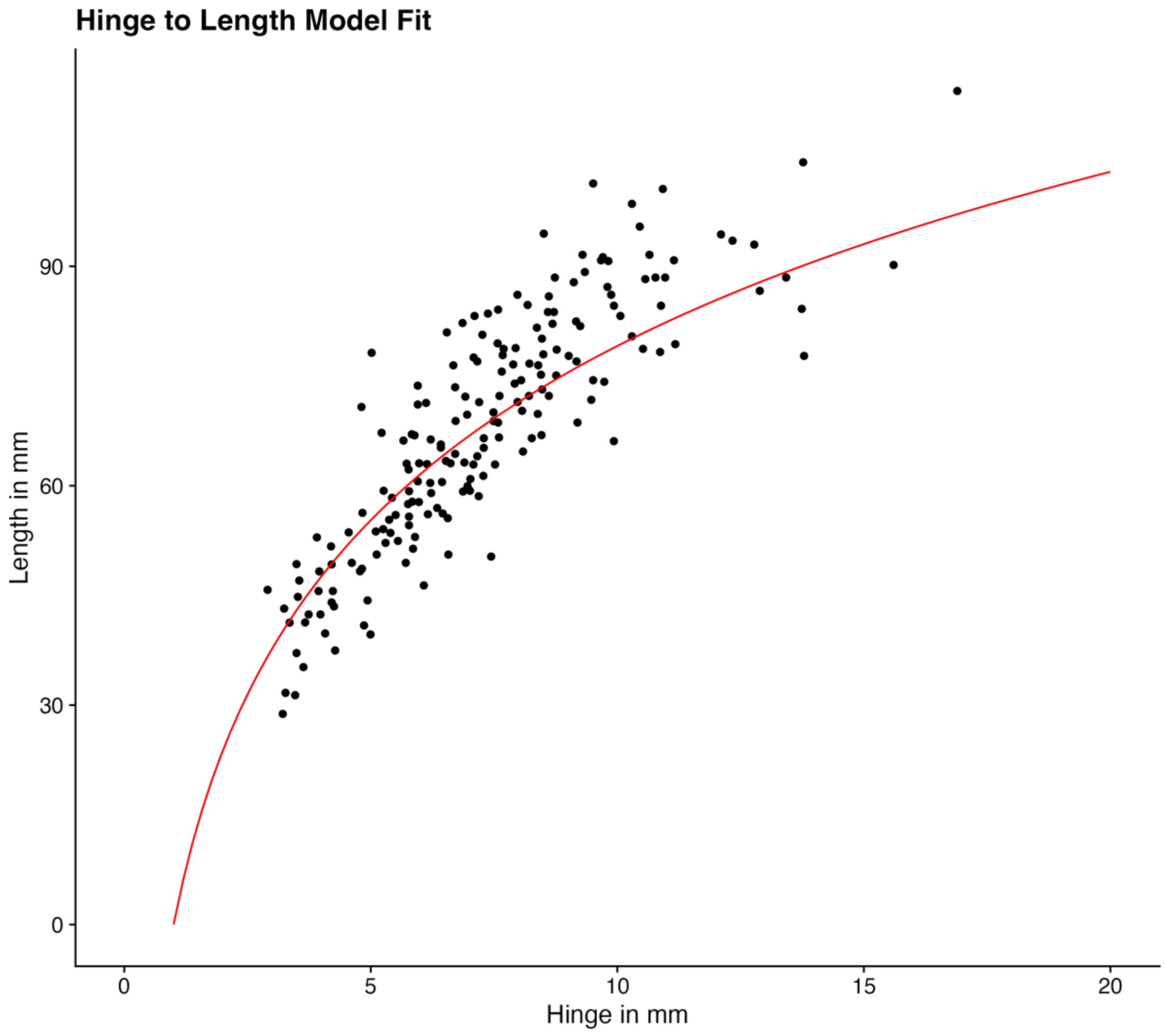

Fig. S5. Relationship between the oyster hinge and length in mm.

Table S1. Summary of archaeological sites and numbers of samples. Key: MM, Middle Mesolithic; LM, Late Mesolithic; EN, Early Neolithic; KON, Kongemose culture; EBK, Ertebølle culture; TRB, Funnel Beaker culture; IGLA, Incremental Growth Line Analysis; *, shell midden; +, cultural layer with shells and/or combination of cultural and natural shells; #, natural shell bank. Note the total shell length measurements to establish the hinge/length growth curve (see Fig. S12).

**Site name (site code)**	**Site type (see caption)**	**Period**	**Culture**	**Approx. dates (cal BCE)**	**No. of hinge/total length measurements**	**No. of oyster shells sampled for IGLA**	**No. of biological age determinations**	**Reference(s) for sclerochronology**	**Key reference(s) for site**
Bjørnsholm (FHM 2911)	*	LM-EN	EBK-TRB	~4900-4000<br>~3900-2700	226	232	125	(3)	(4)
Brovst (FHM 1586)	*	MM-LM	EBK	~4490-4000	22	22	13	(3)	(5)
Dyngby I (FHM 3954)	+	LM-EN	EBK-TRB	~4260-3800	33/56	33	25	(2)	
Dyngby III (FHM 4339)	+	LM	EBK	~5060-4160	19	56	18	(3)	(6)
Ertebølle (FHM 2168)	*	LM	EBK	~4990-4000	139	167	94	(3)	(7, 8)
Ertebølle (VMÅ 2185)	*	LM	EBK	~5400-4000	22	23	20	This study	
Eskelund (FHM 1116)	*	LM	EBK	~3970	20	20	16	(2)	(2)
Eskilsø SØ (MFG 158/99)	*	LM-EN	EBK-TRB	~4300-4000<br>~3900-3500	119	130	110	(3, 9)	(9)
Havnø (FHM 4014)	*	LM-EN	EBK-TRB	~5250-4000<br>~3900-2600	131/13	154	113	(2, 3, 10)	(3, 11)
Hjarnø Sund (FHM 5184)	*	MM-LM	KON-EBK	~5550-4450	159	159	113	This study	(12–14)
Hjarnø Vesterhoved (FHM 5948)	*	MM-LM	KON-EBK	~5460-5120	37	40	32	This study	(15)
Holmegård (FHM 1532)	*	LM	EBK	~5500-4240	33	46	14	(3)	(16)
Krabbesholm II (FHM 4383)	*	LM-EN	EBK-TRB	~5200-4000<br>~3900-3380	397	261	185	(10, 17, 18)	(19–21)
Lystrup Enge (FHM 2718)	*	MM-LM	KON-EBK	~5660-4000	/14			(2)	(22)
Norsminde (FHM 2911)	*	LM-EN	EBK-TRB	~4780-4000	668/71	121	100	(2, 10, 23)	(2, 20, 24)

				~3900-2600					
Tybrind Vig (FHM 2033)	#	LM	EBK	~5400-4000	82	83	68	This study; (3)	(25)
Vængesø III (FHM 4428)	*	LM	EBK	~4500-4000	21	21	12	(3)	(16)
Vestre Strandallé (FHM 6218)	*	LM	EBK	~5400-5000	49	51	38	This study	
Visborg (FHM 3933)	*	EN	TRB	~3900-2980	292/34	295	187	(2, 3)	(26–31)
Visborg (ÅHM 6814)	*	LM-EN	EBK-TRB	~4460-3380	183	193	141	This study	
**Totals**					**1964/188**	**2107**	**1424**		

Stratigraphic sequences for all sites with stratigraphic information

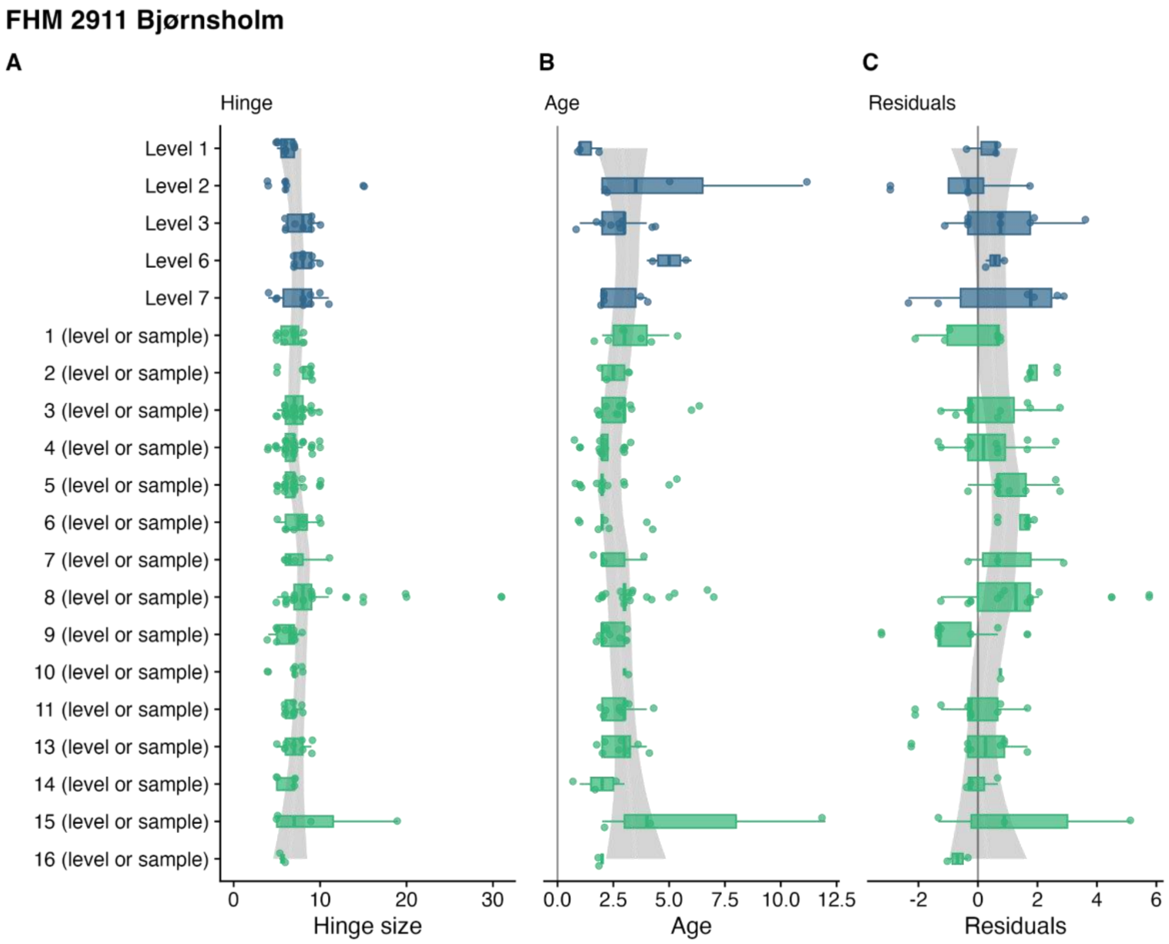

Fig. S6. Stratigraphic sequence of hinge, age and residual data. Mesolithic and Neolithic layers are shown in green and blue respectively.

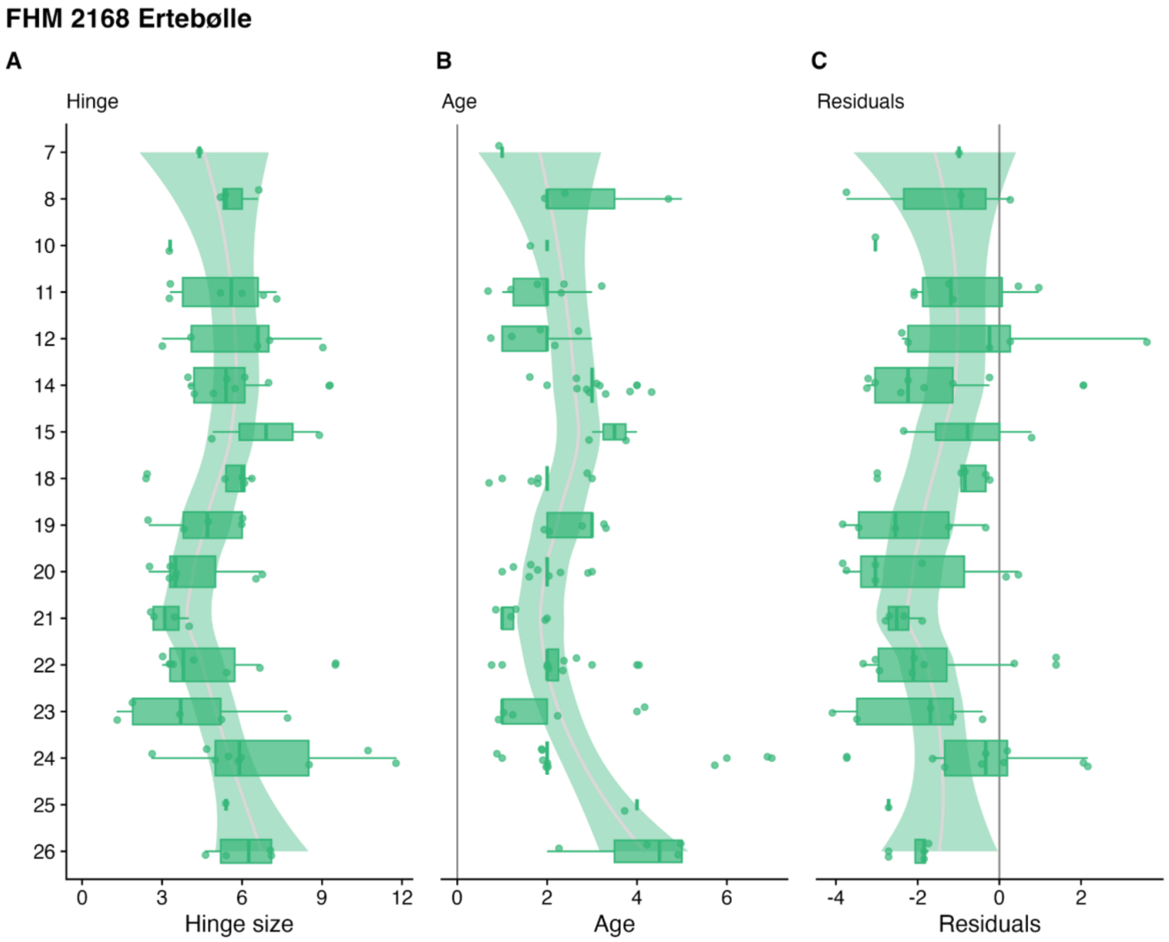

Fig. S7. Stratigraphic sequence of hinge, age and residual data. Mesolithic layers are shown in green.

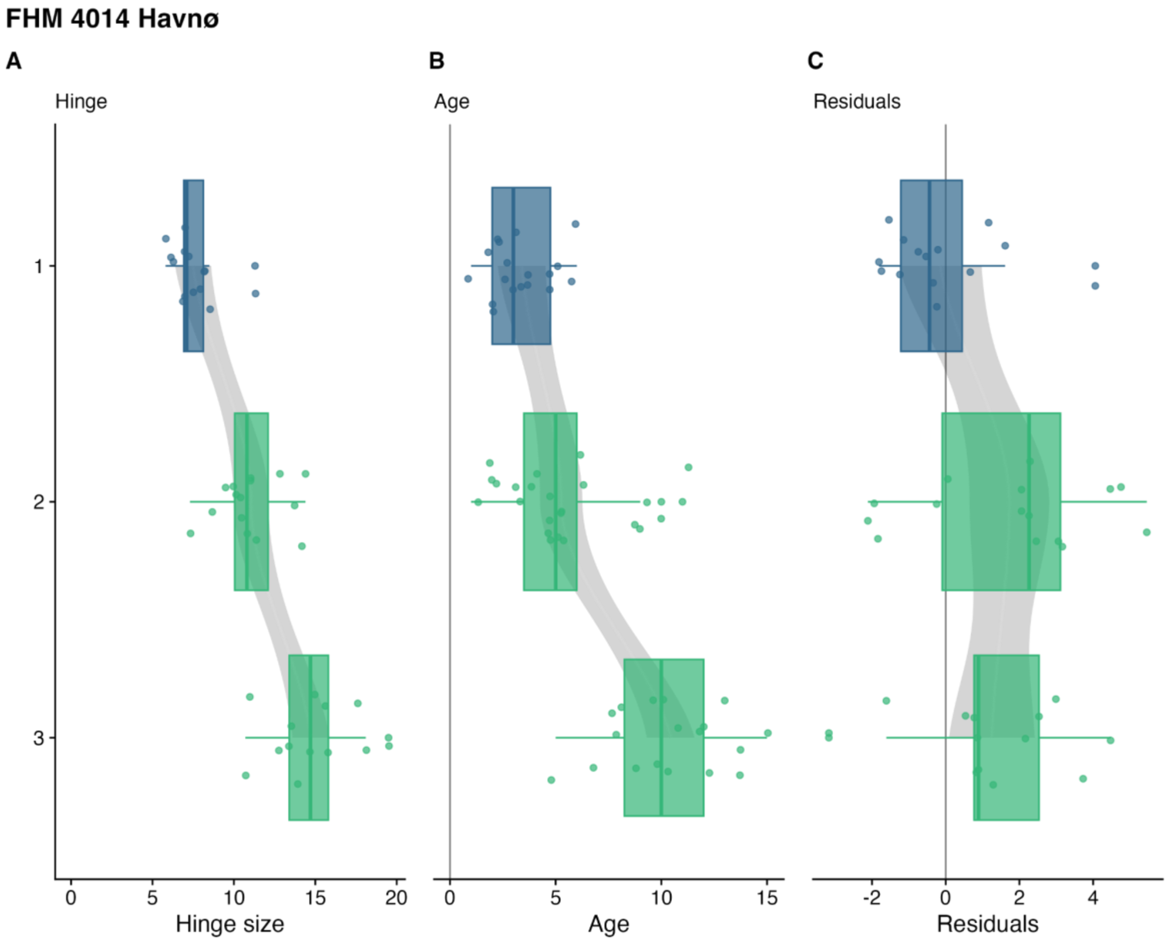

Fig. S8. Stratigraphic sequence of hinge, age and residual data. Mesolithic and Neolithic layers are shown in green and blue respectively.

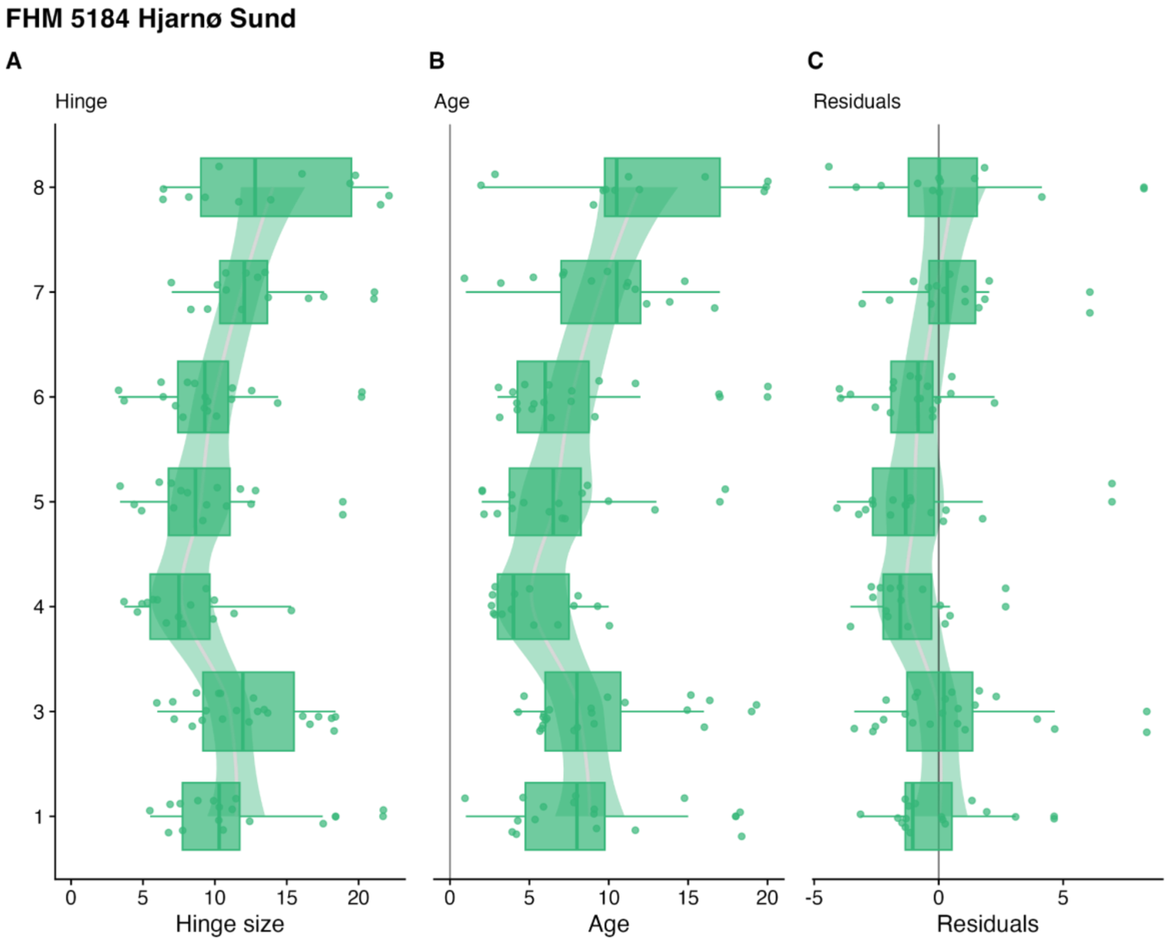

Fig. S9. Stratigraphic sequence of hinge, age and residual data. Mesolithic layers are shown in green.

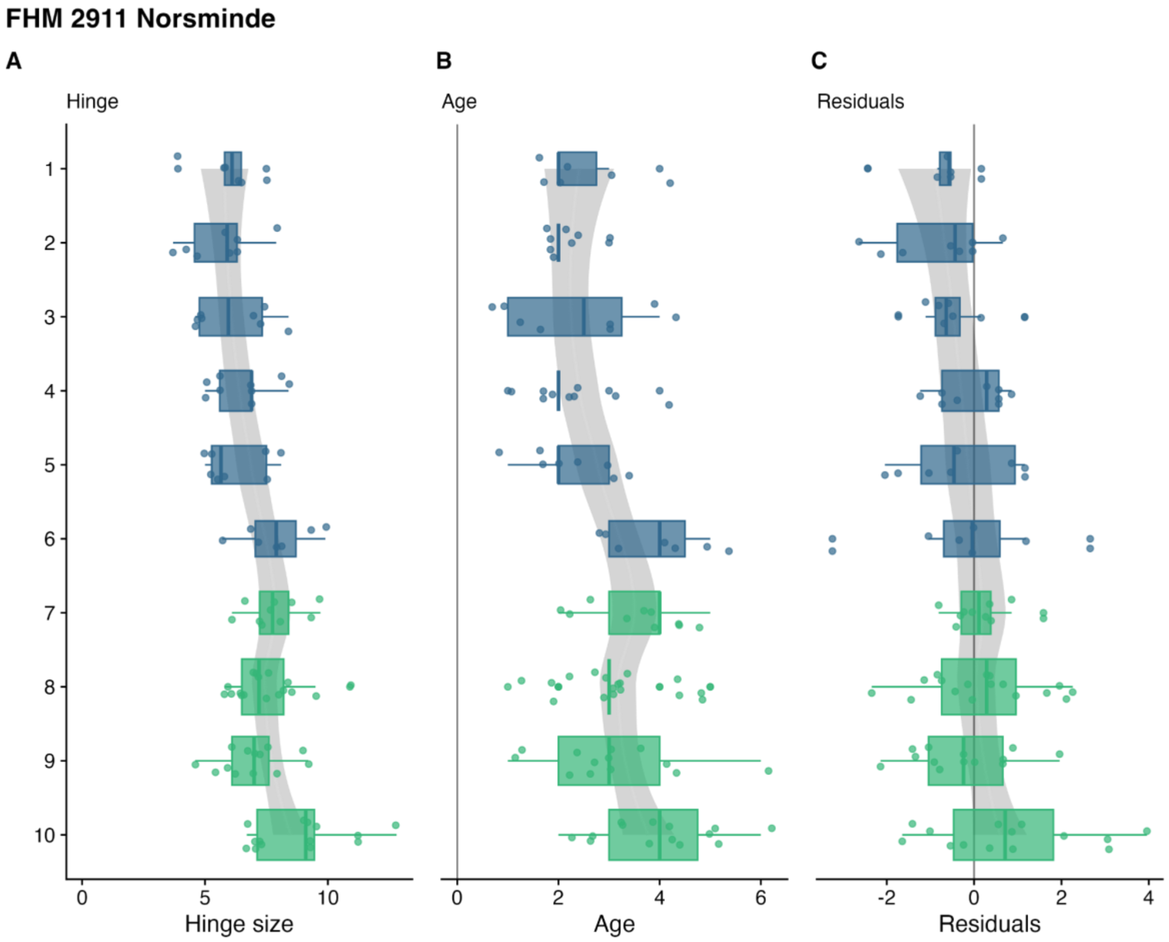

Fig. S10. Stratigraphic sequence of hinge, age and residual data. Mesolithic and Neolithic layers are shown in green and blue respectively.

ÅHM 6814 Visborg

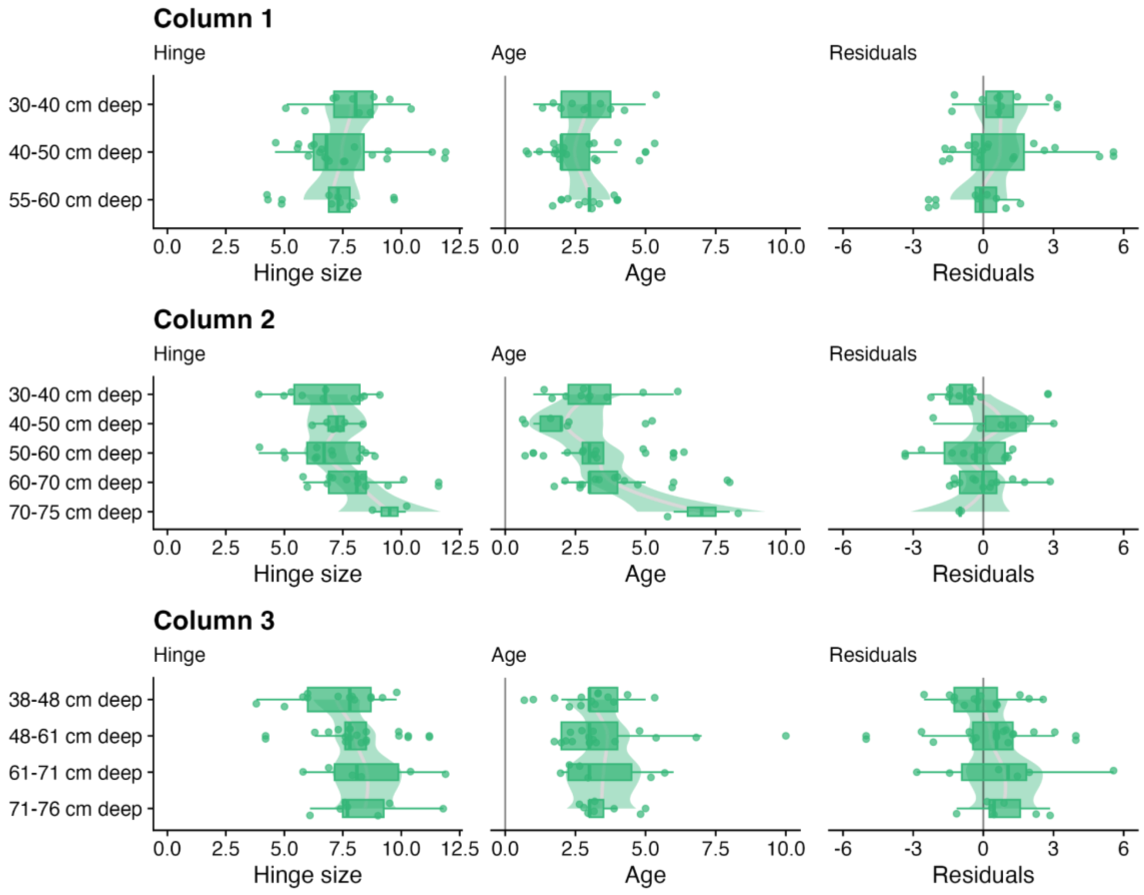

Fig. S11. Stratigraphic sequence of hinge, age and residual data. Mesolithic layers are shown in green.

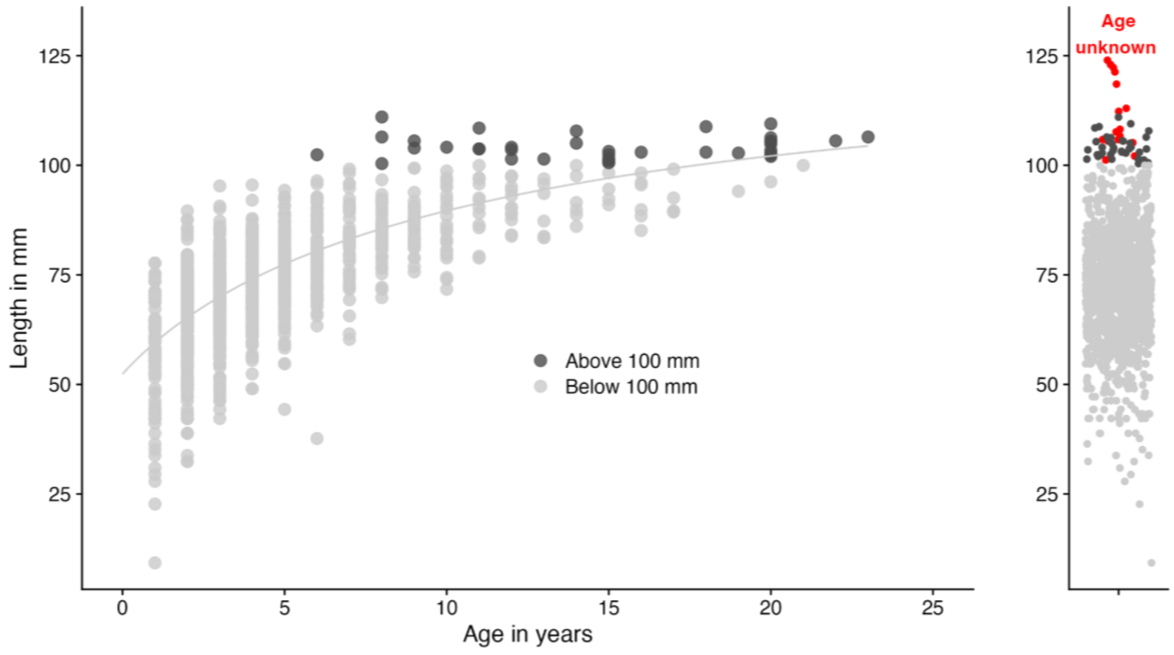

Fig. S12. Top specimens of collections (here using an arbitrary cut off of 100 mm) which most likely represent less harvested parts of the oyster population and best represent the broodstock for the commonly harvested near-shore parts of the oyster reef. The specimens in black have ages of up to 23 years and grow to over 100 mm within only 6 years. Specimens in red do not have reliable age data but still show sizes of up to 124 mm.

Supplementary Information References

1. H. K. Lotze, Radical changes in the Wadden Sea fauna and flora over the last 2,000 years. *Helgol. Mar. Res.* **59**, 71–83 (2005).
2. N. Milner, *Incremental growth of the European oyster Ostrea edulis: Seasonality information from Danish kitchenmiddens* (BAR Publishing, 2002).
3. H. Robson, *Evaluating the change of consumption and culinary practices at the transition to agriculture: a multi-disciplinary approach from a Danish kitchen midden* (PhD Thesis, 2015).
4. S. H. Andersen, Bjørnsholm. A stratified køkkenmødding on the central Limfjord, north Jutland. *J. Dan. Archaeol.* **10**, 59–96 (1991).
5. S. H. Andersen, Brovst, en kystboplads fra ældre stenalder. *Kuml* **19**, 67–90 (1969).
6. S. H. Andersen, Aktivitetspladser fra Ertebølletid. *Kuml* **53**, 9–43 (2004).
7. S. H. Andersen, E. Johansen, Ertebølle revisited. *J. Dan. Archaeol.* **5**, 31–61 (1986).
8. K. S. Petersen, The Ertebølle “køkkenmødding” and the marine development of the Limfjord, with particular reference to the molluscan fauna. *J. Dan. Archaeol.* **5**, 77–84 (1986).
9. H. K. Robson, S. A. Sørensen, E. Laurie, N. Milner, “Incremental growth line analysis of the European oyster (*Ostrea edulis*, Linnaeus, 1758) from the kitchen midden at Eskilsø, Denmark” in *Foraging Assemblages Volume 2*, D. Borić, D. Antonović, B. Mihailović, Eds. (Serbian Archaeological Society and The Italian Academy for Advanced Studies in America, Columbia University, 2021), pp. 404–409.
10. N. Milner, “Human impacts on oyster resources at the Mesolithic–Neolithic transition in Denmark” in *The Archaeology and Historical Ecology of Small Scale Economies*, V. D. Thompson, J. C. Waggoner, Eds. (University Press of Florida, 2013), pp. 16–39.
11. S. H. Andersen, A report on recent excavations at the shell midden of Havnø in Denmark. *Mesolithic Miscellany* **19**, 3–6 (2008).
12. P. M. Astrup, *et al.*, Underwater shell middens: Excavation and remote sensing of a submerged Mesolithic site at Hjarnø, Denmark. *J. Isl. Coast. Archaeol.* **15**, 457–476 (2020).
13. J. S. Larsen, *et al.*, From oysters to cockles at Hjarnø Sund: Environmental and subsistence changes at a Danish Mesolithic site. *Radiocarbon* **60**, 1507–1519 (2018).
14. C. Skriver, P. Borup, P. M. Astrup, “Hjarnø Sund: An eroding Mesolithic site and the tale of two paddles” in *Under the Sea: Archaeology and Palaeolandscapes of the Continental Shelf*, G. N. Bailey, J. Harff, D. Sakellariou, Eds. (Springer International Publishing, 2017), pp. 131–143.
15. P. M. Astrup, *et al.*, A drowned Mesolithic shell midden complex at Hjarnø Vesterhoved, Denmark and its wider significance. *Quat. Sci. Rev.* **258**, 106854 (2021).
16. S. H. Andersen, *Vængesø and Holmegaard: Ertebølle Fishers and Hunters on Djursland* (Aarhus University Press, 2018).

17. N. Milner, Seasonality Studies and Palaeoenvironmental Analysis of Oysters from the Danish Kitchen Midden of Krabbesholm. [Preprint] (2001).

18. N. Milner, E. Laurie, Preliminary report on the seasonality of shellfish gathering and midden composition for the site of Krabbesholm. [Preprint] (2006).

19. S. H. Andersen, Køkkenmøddingerne ved Krabbesholm. *Ny forskning i stenalderens kystbopladser. Nationalmuseets Arbejdsmark,* 151–171 (2005).

20. E. M. Laurie, *An Investigation of the Common Cockle (Cerastoderma edule (L)): Collection practices at the kitchen midden sites of Norsminde and Krabbesholm, Denmark* (BAR Publishing, 2008).

21. N. Nielsen, "Marine molluscs in Danish Stone Age middens: A case study on Krabbesholm II" in *Early Human Impact on Megamolluscs*, A. Antczak, R. Cipriani, Eds. (BAR Publishing, 2008), pp. 157–167.

22. S. H. Andersen, Ertebøllebåde fra Lystrup. *Kuml* **39**, 7–38 (1994).

23. N. Milner, E. Laurie, "Coastal perspectives on the Mesolithic Neolithic transition" in *Mesolithic Horizons. Papers presented at the Seventh International Conference on the Mesolithic in Europe, Belfast 2005*, S. McCartan, R. Schulting, G. Warren, P. Woodman, Eds. (Oxbow Books, 2009), pp. 134–139.

24. S. H. Andersen, Norsminde: A "køkkenmødding" with Late Mesolithic and Early Neolithic occupation. *J. Dan. Archaeol.* **8**, 13–40 (1989).

25. S. H. Andersen, *Tybrind Vig: Submerged Mesolithic settlements in Denmark* (Aarhus University Press, 2013).

26. S. H. Andersen, The Stone Age Coast. *Maritime Archaeology Newsletter from Roskilde Fjord* **11**, 11–13 (1998).

27. S. H. Andersen, Fjorden i oldtiden. *Geologi Nyt Fra Geus* **4**, 10–11 (1999).

28. S. H. Andersen, Fisker og bonde ved Visborg. *Vor skjulte kulturarv. Arkæologi under overfladen. Det Kongelige Nordiske Oldskriftselskab/Jysk Arkæologisk Selskab, Højbjerg*, 42–43 (2000).

29. S. H. Andersen, Visborg. *Maritime Archaeology Newsletter from Roskilde Fjord* **13**, 36–37 (2000).

30. S. H. Andersen, Visborg 2001. *Maritime Archaeology Newsletter from Roskilde Fjord* **17**, 23–27 (2001).

31. S. H. Andersen, Visborg køkkenmøddingen. *Maritime Archaeology Newsletter from Roskilde Fjord* **18**, 9 (2002).
